# Periodic patterning of the *Drosophila* eye is stabilized by the diffusible activator Scabrous

**DOI:** 10.1038/ncomms10461

**Published:** 2016-02-15

**Authors:** Avishai Gavish, Arkadi Shwartz, Abraham Weizman, Eyal Schejter, Ben-Zion Shilo, Naama Barkai

**Affiliations:** 1 Department of Molecular Genetics, Weizmann Institute of Science, Rehovot 76100, Israel; 2 Sackler Faculty of Medicine, Geha Mental Health Center, Felsenstein Medical Research Center, Rabin Medical Center, Tel Aviv University, Bellinson Campus, Petah Tiqva 49100, Israel

## Abstract

Generation of periodic patterns is fundamental to the differentiation of multiple tissues during development. How such patterns form robustly is still unclear. The *Drosophila* eye comprises ∼750 units, whose crystalline order is set during differentiation of the eye imaginal disc: an activation wave sweeping across the disc is coupled to lateral inhibition, sequentially selecting pro-neural cells. Using mathematical modelling, here we show that this template-based lateral inhibition is highly sensitive to spatial variations in biochemical parameters and cell sizes. We reveal the basis of this sensitivity, and suggest that it can be overcome by assuming a short-range diffusible activator. Clonal experiments identify Scabrous, a previously implicated inhibitor, as the predicted activator. Our results reveal the mechanism by which periodic patterning in the fly eye is stabilized against spatial variations, highlighting how the need to maintain robustness shapes the design of patterning circuits.

## Abstract (web summary)

Patterning in the *Drosophila* eye is achieved by a series of signalling cascades over several cell distances. Here Gavish *et al.* model lateral inhibition in the developing eye to understand how developmental noise refines such patterning, identifying a novel activator required for buffering spatial variability.

## Graphical abstract

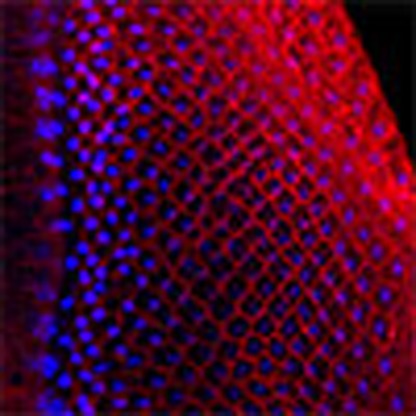

During the development of multicellular organisms, uniform fields of cells are patterned into distinct cell types that are specified in well-defined positions. Patterning depends on biomolecular circuits of interacting signalling molecules that propagate information between cells. Since the patterned regions often extend over many cells, the biochemical parameters defining patterning circuit dynamics may vary across this field. In this study, we examine how such spatial variations have an impact on the design and function of patterning circuits, generating long-range periodic arrangements of selected cells.

A precise periodic selection of photoreceptor cells is critical for the proper formation and function of the *Drosophila* eye. The fly eye is composed of hundreds of highly ordered individual light-sensing units termed ommatidia^1^. The ordered arrangement of the ommatidia is defined at the larval stage, when a subset of cells in the eye imaginal disc differentiate into photoreceptor precursor cells and begin expressing the transcription factors Atonal (Ato) and Senseless (Sens). A differentiation wave sweeps across the eye-disc mono-layered epithelium from the posterior to the anterior side, marked by a visible indentation of the tissue (the morphogenetic furrow, MF)^2^,^3^. *ato*-expressing cells are selected sequentially, concomitant with wave propagation^4^,^5^,^6^. Wave propagation depends on the secretion of Hedgehog (Hh) by posteriorly differentiated cells, which triggers the production and secretion of Decapentaplegic (Dpp) by all cells in the MF^3^,^7^,^8^,^9^. These activators diffuse across the tissue and initiate *ato* expression when reaching uninhibited cells, thereby prompting subsequent differentiation of more anterior cells^2^,^3^,^8^,^10^. While the activation wave triggers cell selection, the periodic pattern depends on the selected cells, producing diffusible inhibitors that prevent selection of nearby cells, ensuring that each differentiated founder cell is surrounded by at least 20 unselected cells that are needed to form the future ommatidium^11^.

Ato is the major transcription factor required for pro-neuronal differentiation. On the arrival of the MF, intermediate-level *ato* expression is first observed in all undifferentiated cells positioned anterior to the furrow. As differentiation proceeds, this uniform *ato* stripe is refined into evenly spaced pro-neural clusters of ∼10 cells. Finally, each cluster is resolved into a single *ato*-expressing cell^10^,^12^.

Previous studies implicated two diffusible inhibitors that are needed for the first stage of stripe refinement into clusters: Scabrous (Sca) and an additional, not yet identified inhibitor that depends on signalling by the epidermal growth factor receptor (Egfr)^13^,^14^. The Notch–Delta pathway also plays a major role in this patterning, activated Notch being a key inhibitor of *ato* expression during the two refinement stages^10^,^15^,^16^,^17^.

Proper definition of cluster size and position is the key for reliable propagation of the periodic pattern of selected cells. The evenly separated clusters in each column serve as a template for the next column: the effective inhibitory circuits formed around each cluster by the secreted inhibitors define the positions of clusters of the subsequently differentiating column by the points of minimum inhibition^5^,^13^,^14^. Any error in cluster position or cluster size will propagate between columns, leading to error amplification. The final stage of cluster refinement ensures the eventual selection of a single cell from each cluster, but cannot retrieve proper patterning when clusters are merged or misplaced.

The ability of this template-based lateral inhibition circuit to produce the periodic pattern observed in the *Drosophila* eye disc was confirmed recently by computer model simulations^18^,^19^. We find, however, that this circuit is inherently non-robust, failing to reproduce patterns in the presence of small variations between cells (noise). This extreme noise sensitively is enhanced when extending the model to simulate selection of pro-neural clusters. Error in cluster size or position rapidly propagates between columns, leading to occasional selections of large elongated clusters (‘catastrophes'), which cannot be corrected at the final refinement stage. We explain the origin of this high noise sensitivity and find that robustness to spatial variability is restored when a short-range diffusible activator is assumed to complement the lateral inhibition circuit. We identify this missing activator as Sca, a previously implicated inhibitor^13^, and provide experimental evidence supporting this role. Our results emphasize how patterning circuits are designed by the need to buffer spatial variations in biochemical parameters.

## Results

### Lateral inhibition is highly sensitive to spatial variations

**Figure 1:**
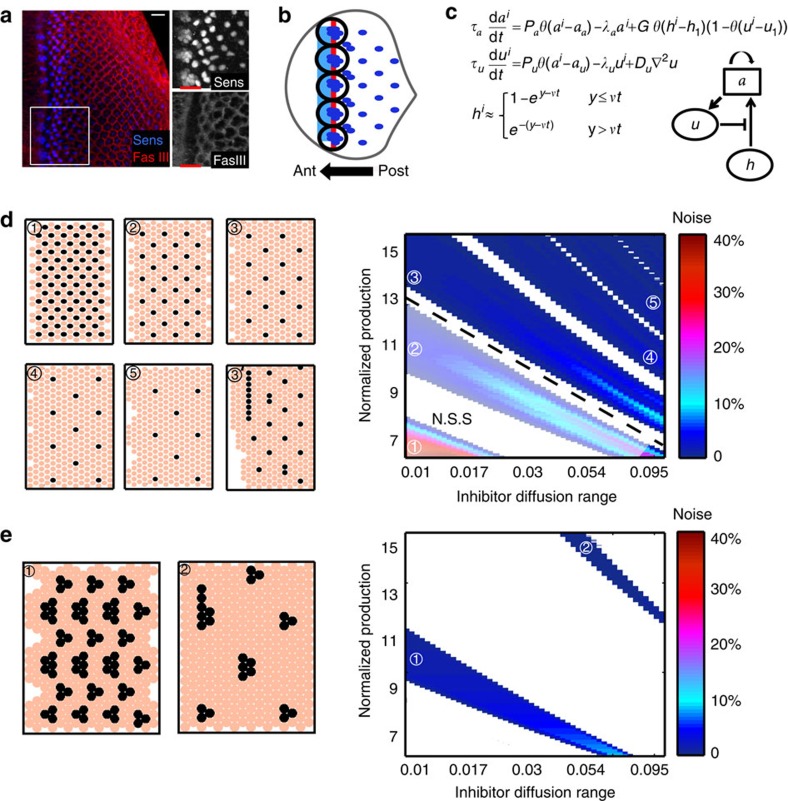
A model of eye disc patterning. Here and in all figures posterior is to the right. (**a**) Confocal image of the developing disc. Cell outlines are visualized with FasIII (red), and differentiating cells with the nuclear differentiation marker Sens (blue). Inset: a magnified view of the boxed region, including and immediately posterior to the MF (red line). Scale bar, 10 μm. (**b**) Schematic representation of *sens* expression. *ato* is first expressed uniformly (blue stripe) anterior to the MF (red line), upon which it is refined into pro-neural clusters and expressed together with *sens*. (**c**) Equations and interactions are shown for the template-based lateral-inhibition patterning model. *h* denotes the long-range activator (representing Hh and Dpp), *a* the cell-autonomous activator (representing Ato and Sens) and *u* the diffusible inhibitor (representing Sca and the Egfr-dependent signal). Simulations are performed by discretizing the equations on a 42 × 42 cell grid, with *h* value approximated by its analytical value (Supplementary equation (12.3)) whose approximation is shown here (*v* represents wave velocity). *τ*_*a*_ and *τ*_*u*_ denote the typical timescales characterizing the dynamics of *a* and *u*, respectively; *λ*_*a*_ and *λ*_*u*_ are the respective degradation rates; *P*_*a*_ and *P*_*u*_ are the respective production rates; *D*_*u*_ is the diffusion constant of *u*. *θ*(*a*) is the Heaviside step function, which is equal to 1 for positive values and zero otherwise. The upper-script indexes (*i*) indicate the grid site and ∇^2^ is the grid two-dimensional Laplacian operator. More details are given in Supplementary Note 1. (**d**,**e**) Parameters defining the inhibition range (effective diffusion, *D*_*u*_, and normalized production rate *P*_*u*_/*u*_1_) were varied systematically, as shown. White regions denote parameter combinations where no periodic solution was found. Colour scale quantifies noise sensitivity by the maximal spatial variations that can be added without pattern failure. We examined patterns of solitary cells (**d**) and of clusters (**e**). Panel 3′ demonstrates pattern failure after adding less than 5% noise to parameters generating the pattern in panel 3. For each parameter configuration, maximal noise is reported after simulating all possible initial conditions. The parameter space enclosed below the dashed line (N.S.S; non-sufficient spacing) yields patterns in which the area surrounding each cluster does not comprise the required 20 cells forming the ommatidium. See Supplementary Methods for details of how noise was defined and Supplementary Table 1 for the parameters used.

The template-based lateral inhibition circuit^18^,^19^ approximates the patterning by considering three effective components: the primary cell-autonomous transcription factor (*a*), an Ato-dependent, short-range diffusible inhibitor (*u*) and a uniform long-range diffusible activator (*h*; Fig. 1a–c). Here the inhibitor *u* simulates the combined function of all diffusible inhibitors, *a* simulates the function of all self-autonomous auto-activators (Ato and Sens), while *h* simulates the function of all long-range diffusible activators (Hh-dependent Dpp signalling).

To approximate the patterning dynamics in the eye disc, we considered a two-dimensional (2D) cell arrangement, with cells positioned on a 42 × 42 hexagonal grid. We begin the differentiation from a pre-pattern (initial condition) of selected cells at the posterior-most region. The size of the clusters in the pre-pattern and the spacing between these clusters ultimately define the initial condition in each simulation. An activation wave *h*(*y, t*) propagates with a velocity *v* from the posterior to the anterior side, and induces *a*^*i*^ expression when its levels at cell position (*x*^*i*^, *y*^*i*^) attain some preset threshold *h*_1_. This linear propagation across the disc reflects the expression pattern of Dpp, which is observed uniformly anterior to the MF^2^,^20^. *a*^*i*^ accumulation leads to its auto-activation (when *a*^*i*^>*a*_*a*_), and to the production of the inhibitor *u*^*i*^ (when *a*^*i*^>*a*_*u*_), which spreads to nearby cells *j* to inhibit *a*^*j*^ expression (when *u*^*i*^>*u*_1_). This model was solved numerically by discretizing the equations in Fig. 1c, and solving them sequentially for each individual cell at subsequent times *t* separated by short time intervals (d*t*=10^−2^), each time calculating the activation level *h*(*y*^*i*^, *t*) according to its analytical approximation (given fully in Supplementary Methods), and using the simulated values of *a*(*x*^*i*^, *y*^*i*^, *t*) and *u*(*x*^*i*^, *y*^*i*^, *t*) (more details are given in Supplementary Notes 1–4). Extending the model by explicitly assuming additional factors performing the same function does not change its qualitative function (see Supplementary Note 5 on adding late *sens* auto-activation).

As was shown before, this minimal system can generate a periodic pattern similar to the one observed in the eye imaginal disc, with single-cell clusters surrounded by 20 unselected cells obtained already at this initial stage of cluster formation, alleviating the need for further cluster refinements^18^. Consistent with previous results, however, this pattern is attained only for a restricted set of parameters, requiring that production rates and diffusion coefficients are properly tuned, and is rapidly lost when changing parameters or when allowing some spatial variations in production rates or diffusion coefficients. To examine whether this lack of robustness is general or specific to the parameters chosen, we searched for parameters that reduce sensitivity to spatial variations. To this end, we systematically varied the cell-specific inhibitor production rates and diffusion coefficients (the two most-sensitive parameters defining the range of inhibition as shown in Supplementary Note 3). Notably, periodic patterns were obtained for some parameters (coloured regions in Fig. 1d,e) but not for others (white regions in Fig. 1d,e), even in the absence of spatial variations. Focusing on the parameters that did produce a periodic pattern, we noted that the model defined different pattern types, classified by the cluster size and/or the intercluster spacing.

To quantify noise sensitivity, we next simulated the model for each parameter set while considering different levels of spatial variability. Spatial variability was introduced by choosing for individual cell production, and diffusion rates from uniform distributions centred at some reference parameters (see Supplementary Fig. 1 on adding noise to more parameters). These reference parameters were chosen to best fit biological knowledge^18^ (see Supplementary Table 1 and Supplementary Methods). For example, the rate by which the cell *i* produced inhibition was chosen from a uniform distribution whose width was 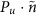, *P*_*u*_ being the global chosen parameter and 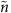 the noise level. We tested all possibilities of initial spacing between clusters in the first column (initial conditions) for each parameter configuration. Noise sensitivity was then defined as the highest level of noise 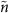, allowing proper patterning on optimization of initial conditions. Specifically, in the phase diagram shown in Fig. 1d, a pattern was considered destroyed if an elongated-shaped cluster of more than four cells was formed (Fig. 1d, plane 3′) or if 5% cases or more of ‘twinning' occurred (two cells or more differentiated adjacently). In the phase diagram shown in Fig. 1e, a pattern was considered destroyed if an elongated cluster with more than 10 cells formed.

As can be appreciated from Fig. 1d,e, all patterns were highly sensitive to even small levels of spatial variations. The only exception was the pattern of tightly spaced one-cell clusters (Fig. 1d, branches 1 and 2). These patterns, however, do not fit the biological setting in the *Drosophila* eye, as the area surrounding each cluster does not comprise the required 20 cells forming the ommatidium (non-sufficient spacing, noted as N.S.S. in Fig. 1d). We conclude that patterning by the template-based lateral inhibition model is highly sensitive to spatial variations and therefore cannot be used to reliably pattern the *Drosophila* eye.

### The origin of noise sensitivity in two dimensions

**Figure 2:**
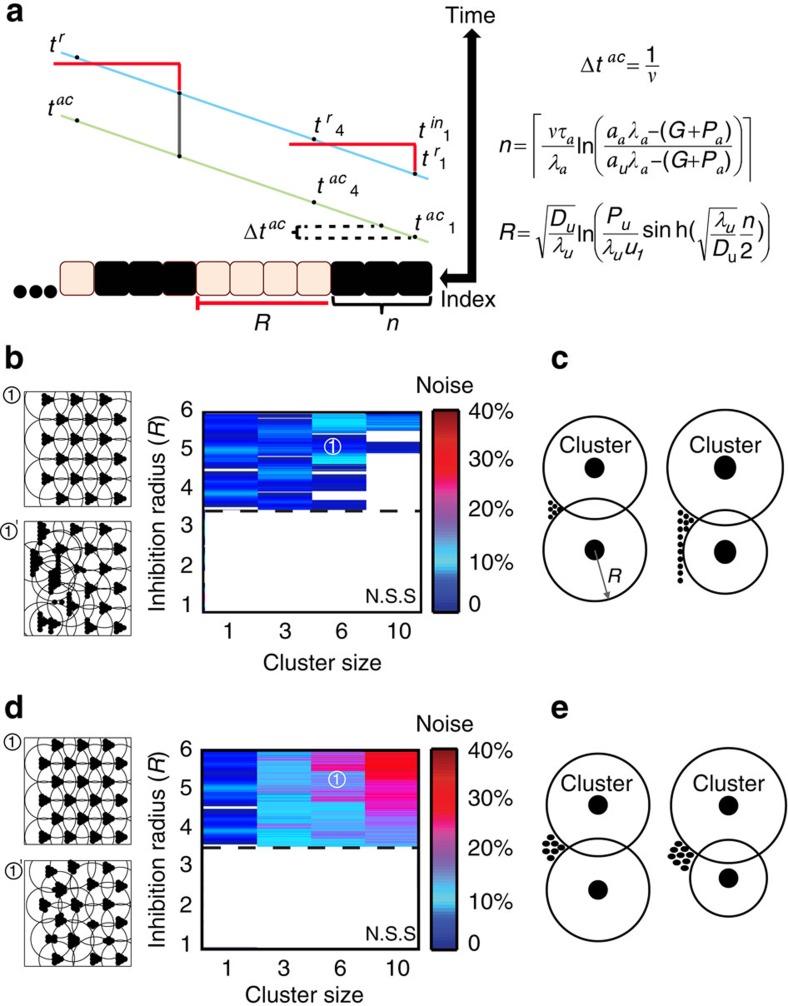
Incompatibility of linear activation propagation and radial inhibition. (**a**) Pattern formation in one dimension. Time is on the vertical axis and cell position on the horizontal axis. Cluster size is defined by the number of cells that become refractory before the first cell in the cluster produces inhibition (red horizontal line). 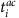 is the time when cell *i* receives sufficient *h* to induce *a* expression, provided that it was not yet inhibited. 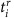 is the time when an activated cell becomes refractory. 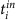 is the time when an activated cell begins secreting the inhibitor *u*. Δ*t*^*ac*^ is the time gap between activation of two adjacent cells. *n* is the size of the cluster. *R* is the distance between clusters. The dependence of those times on the model parameters is derived in Supplementary Note 2. (**b**) Shown is the noise sensitivity of the simplified model in two dimensions for different values of inhibition radii and cluster sizes. Simulations were performed on a grid by sequentially selecting clusters of the desired size and drawing inhibition radii around them. Noise sensitivity was defined by the maximal noise level that can be introduced before pattern failure, for optimized initial conditions as we did in Fig. 1c. Noise was introduced by selecting each inhibition radius from a uniform distribution that was centred at the indicated values *R* and whose width was 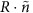, 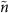 defining the noise level. A pattern of cluster size 1 was considered destroyed when a cluster of size larger than 3 was formed. Similarly, a cluster of 3, 6 and 10 was considered destroyed when clusters of 6, 10 and 15 formed, respectively. All simulations were run until cell differentiation reached the end of the grid. N.S.S. stands for non-sufficient spacing as in Fig. 1d. (**c**) Selection of a long, uninhibited cell line (catastrophe) is the main source of noise sensitivity. See Supplementary Fig. 2 for more details. (**d**) Same as (**b**) for the extended model including an activator. In addition to adding noise to the inhibition radii, noise was added to the activation radii in a similar manner. Pattern failure was determined as in **b**. (**e**) Since cluster size is now defined by the short-range activator, rather than propagation of *h*, sensitivity to catastrophes is reduced.

To better understand the high noise sensitivity of the template-based lateral inhibition model, we examined its dynamics using analytical approximations. For simplicity, consider first pattern progression in a one-dimensional (1D) space (Fig. 2a). At time *t*=0, the activation wave *h* begins to propagate from the posterior to the anterior side, activating adjacent cells with a time delay that depends on *h* velocity. The first cell encountering sufficient *h* will begin producing the cell-autonomous activator *a.* Once *a* accumulates above some threshold, the cell will be ‘selected': *a* production in this cell becomes independent of *h* and of possible inhibitory signals. When attaining a second (higher) threshold, the cell will begin producing the inhibitor *u* that diffuses rapidly over some distance to inhibit *a* expression in cells that were not yet selected.

Cluster size *n* is determined by the number of cells that are selected before the first cell within the cluster begins expressing the inhibitory signal *u*. It therefore depends on the velocity of the differentiation wave, and the time elapsed from selection to inhibitor production by an individual activated cell (Fig. 2a). The spacing between clusters, on the other hand, is determined by the distance *R* over which the secreted inhibitor *u* is effective, and therefore depends primarily on its production, diffusion and degradation rates. This simple analysis suggests that patterning is robust and should be achieved over a wide range of parameters. Indeed, when simulating this 1D approximation, patterns with the predicted properties are easily obtained and are practically insensitive to spatial variations (introduced by choosing the inhibition range from a uniform distribution of width 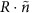
*, R* being the mean range and 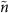 the noise level)^19^.

In contrast, in two dimensions, simulations of this simplified model became highly sensitive to noise, similar to our results with the full model (Fig. 2b). We noted that the critical distinction between the 1D and 2D dynamics resides in the symmetry: in the 1D case, propagations of the activator *h* and of the inhibitor *u* are overlapping in space. In contrast, in 2D, *h* propagated anteriorly as a line (uniform along the length of the lattice), while inhibition forms circles around each cluster. Consequently, the number of cells selected at each column is different, depending on the intersection of two adjacent inhibition circles (Fig. 2b,c). This is critical, as it is now possible that following a small error, a large region that is not constrained by the inhibitory circles will be activated by the propagating *h* signal, leading to the recruitment to the cluster of a large number of cells, or even a full column. Such so-called catastrophes are readily obtained even in the presence of small levels of heterogeneity and are practically impossible to correct. Once a line template is generated, it will continue to propagate as a line, and the hexagonal pattern cannot be restored by the subsequent lateral-inhibition process^18^ (Supplementary Fig. 2).

### A short-range diffusible activator can buffer noise

Catastrophes, as described above, are inherent to the model of template-based lateral inhibition in two (or higher) dimensions and result from the difference in symmetry between the radial inhibition circles and the linear propagating wave. We reasoned that a stabilizing factor, which propagates the activation signal radially over short distances, might help to overcome such catastrophes, thereby contributing to the robustness of pattern formation. Notably, although working over a short range, such a diffusible activator will act differently from a cell-autonomous activator such as Sens that only changes the effective time of *ato* accumulation (Supplementary Fig. 3a).

To examine the noise sensitivity of this extended model, we repeated our simulations as described in Fig. 2d. Noise was now added to the parameters defining the inhibition circle surrounding each cluster (as in Fig. 2b) and to the activation circle surrounding the posterior-most cell (or cells) in each cluster. Indeed, the extended model was significantly less sensitive to spatial variations. This increased robustness stems from the fact that cluster boundaries are defined by an activating signal propagating with the same radial symmetry as the inhibitory one, thereby protecting the pattern against catastrophes (Fig. 2e).

**Figure 3:**
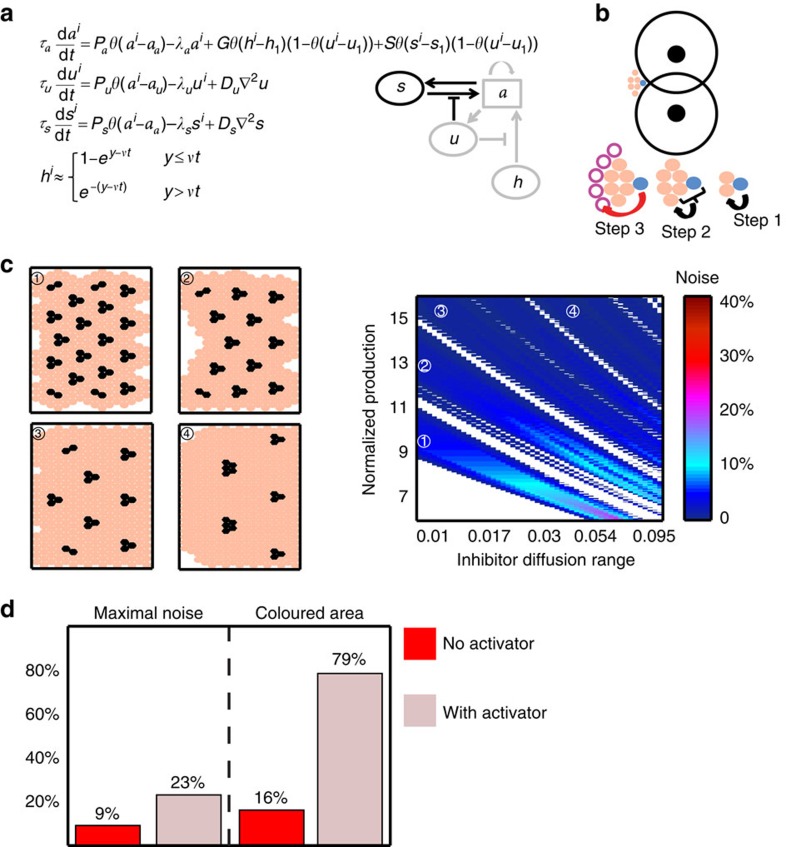
An extended model including a short-range activator. (**a**,**b**) The extended model including a diffusible activator *s*. Shown are the model's equations, the scheme of the interactions and illustration of the activating dynamics. *τ*_*s*_, *P*_*s*_, *λ*_*s*_, *D*_*s*_ are the respective timescale, production rate, degradation and diffusion constants of the short-range activator added to the template-based lateral-inhibition patterning model. In **b**, black arrows depict progression of the short-range activator signal and the red arrow depicts that of the inhibitor. (**c**) Reduced sensitivity of the model to spatial heterogeneity. Sensitivity analysis for the extended model is shown, similar to the one conducted for the original model. See Supplementary Note 6 and Supplementary Fig. 3 for details and Supplementary Tables 1 and 2 for parameters. (**d**) Quantitative comparison of the robustness with or without the addition of the short-range activator. Left panel compares the maximal noise that could be added in simulations shown in **c** to that in simulations in Fig. 1e (no activator). Right panel compares the area of the coloured regions in these simulations, where patterns could be obtained.

We next extended the model in Fig. 1c to examine the ability of a diffusible activator to enhance patterning robustness when simulating the full equations. The model was complemented by the additional variable *s* simulating the activator (Fig. 3a,b). Similar to the inhibitor *u*, *s* induction began when *a* reached some threshold. To be effective, the threshold for *s* production was assumed to be lower than that required for inducing the inhibitor *u,* so that the activator was induced before the inhibitor. *s* was then allowed to diffuse; however, its diffusion range was assumed to be smaller than that of *u.* It reached adjacent cells faster than the global activator *h* and was sufficient by itself to induce *a* expression. Therefore, cluster size was now determined by the range of *s* diffusion, rather than the velocity of *h*, whose effective role was now confined to the initiation of new clusters (more details are given in Supplementary Note 6).

To examine the noise sensitivity of this extended model, we repeated our simulations as described in Fig. 3c. Noise was now added also to the production rate and diffusion coefficient of *s* (in addition to adding noise to all other parameters as in Fig. 1d,e. See Supplementary Fig. 3b on adding noise to all parameters). Indeed, similar to the simplified model in Fig. 2d, the extended model was significantly less sensitive to spatial variations in parameters, and was capable of generating patterns of well-separated clusters over a significantly wider range of parameters (Fig. 3c,d). Furthermore, it now buffered changes in furrow velocity, consistent with experimental reports^7^. The protection against catastrophes provided by a short-range diffusible activator also allows defining clusters of larger sizes, further buffering noise in inhibitor production.

### Sca as the predicted diffusible activator

Our analysis predicts that a short-range activator functions in conjunction with a longer-range inhibitor to enable robust patterning of the eye disc. Such an activator, however, was not included in previous descriptions of the system. In contrast, previous studies implicated two inhibitors in this process: the secreted protein Sca and an unknown inhibitor induced by Egfr signalling^13^,^14^. We reasoned that multiplicity of inhibitors is not necessary, and therefore the action of one of them may have been misinterpreted. Of the two, Sca production is rapid and appears before Egfr activation^13^,^21^, consistent with our demand for rapid activation followed by a delayed repression. We therefore asked whether Sca is in fact an activator, rather than an inhibitor of *ato* expression.

In wild-type (WT) discs, the eventual pattern following cluster refinement consists of single *ato*-expressing cells that are well separated. In contrast, in *sca-*mutant discs, this eventual pattern is disturbed with many instances of adjacent cells expressing *ato.* These so-called *twinnings* follow unsuccessful cluster formation, as the initial clusters in this background are often merged, with *ato* now expressed by a significantly larger number of cells^13^. This increase in *ato-*expressing cells upon *sca* depletion naturally implicates Sca as an inhibitor of *ato* expression. However, biochemical studies indicate differently: Sca was shown to repress Notch, and Notch was shown to act as the main inhibitor of *ato* expression during the stages of cluster formation and refinement^10^,^15^,^16^,^17^,^22^. Therefore, these biochemical lines of evidence implicate Sca as an activator, rather than an inhibitor of cluster formation. We reasoned that the apparent discrepancy between the biochemical function of Sca and the phenotypes of *sca-*depleted discs might not reflect its immediate phenotype but is due to error propagation, as described in our simulations. Specifically, if Sca is an activator, its depletion is expected to first decrease cluster size; however, error propagation would result in ‘catastrophes' leading to larger clusters that will not be refined properly.

**Figure 4:**
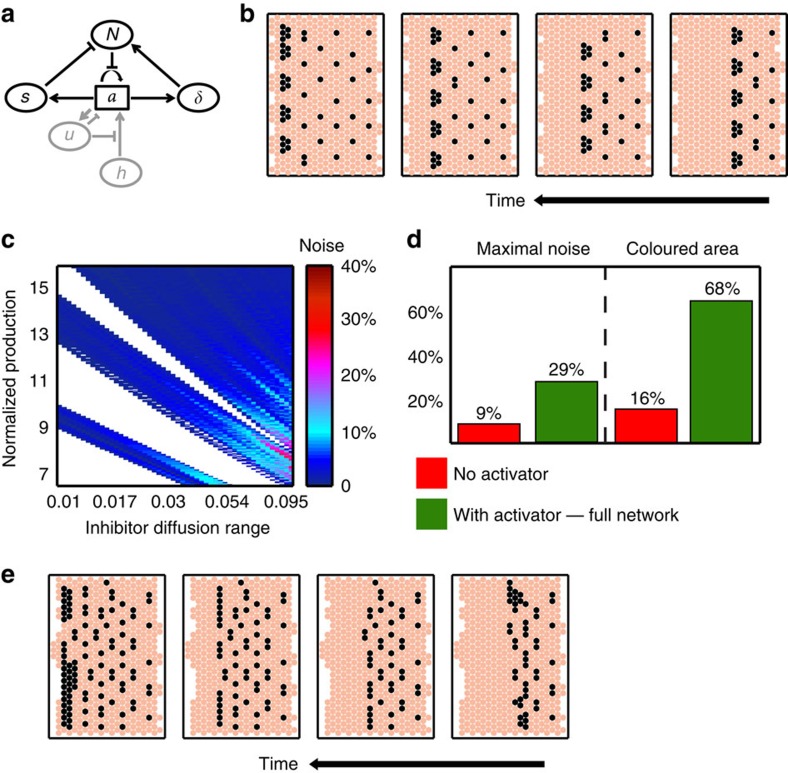
Simulating *sca* function as a competitive inhibitor of Notch activity. (**a**) Model scheme of the full network including Notch and Delta. Model equations are given in the Methods and in Supplementary Note 7. (**b**) Introducing the Notch–Delta interactions enables simulating cluster formation together with cluster refinement. Simulations of the same eye disc are shown at different times (indicated by horizontal arrow), capturing the different stages in eye development. (**c**,**d**) An analysis of the sensitivity of the model to spatial heterogeneity for the extended model. Quantitative comparison of the robustness with or without the addition of the short-range activator is shown in **d**. Left panel compares the maximal noise that could be added in simulations of the full network shown in **c** to that in simulations in Fig. 1e (no activator). Right panel compares the area of the coloured regions in these simulations, where patterns could be obtained. Parameters can be found in Supplementary Table 3. (**e**) In *sca loss of function* mutants, the first distinct phenotype is impaired cluster formation because of increased sensitivity to spatial heterogeneity. The second distinct phenotype is reflected in many cases of impaired cluster refinement leading to a high frequency of twining. Simulations are shown at different times, similar to those shown in **b**.

To more rigorously define the expected phenotype of *sca-*depleted discs, we extended our model to include the biochemically defined role of Sca as a Notch inhibitor. Notch plays a dual function in this dynamics. At the very initial stages, before cluster formation, Notch signalling promotes *ato* expression indirectly in a uniform stripe of cells anterior to the MF by inhibiting the *ato* inhibitors Hairy and Emc (refs 10, 15). Since we are interested in the stage of cluster formation, we did not include this interaction in our model. Rather, we focus on the later stages of cluster formation and refinement, where activated Notch acts as a direct inhibitor of *ato* (Fig. 4a, for full equations see Methods and Supplementary Note 7)^17^. Notably, as lateral inhibition by the Notch–Delta pathway is critical during the phase of cluster refinement, we did not consider this stage in our previous simulation. Including this final step in our model enabled us to fully simulate the dynamics, first following the stage of cluster formation and then the stage of cluster refinement.

We complemented our model by two additional parameters simulating the Notch–Delta pathway. Activated (Delta-bound) Notch was assumed to inhibit *ato* expression, and binding of Sca to Notch prevented Delta binding and thereby led to an effective Notch inhibition (Fig. 4a). The dynamics of *a* now depends explicitly on the levels of *a, u* and activated Notch (Delta-bound), with *h* inducing *a* activity, while both *u* and activated Notch repress *a* expression. As before, *a* begins to be produced once *h* levels increase above some threshold and it begins to induce its own expression when exceeding some threshold. Cells in which *a* reaches a second, higher threshold become refractory to inhibition by *u* signalling, and are thereby selected to a cluster. Notably, at this stage, *a* expression can still be inhibited by activated Notch. The Notch ligand, Delta, is also activated by *ato.* Once one of the *ato-*expressing cells produces sufficient Delta, it inhibits all of its neighbouring cells, thereby remaining the only selected cell. Sca acts as an effective activator of *ato* expression in this model by out-competing Delta, and therefore reducing the level of the activated Notch. The increased *ato* expression by *sca* further contributes to the rapid accumulation of Delta, facilitating the lateral-inhibition dynamics of the last refinement when one of the cluster cells inhibits all of its neighbours and remains the only selected cell (Fig. 4b).

We first verified that this double-negative configuration, in which Sca limits the inhibitory function of Notch, enables robust patterning in the same manner as was observed when Sca was simulated as a direct activator (Fig. 4c,d). We then asked whether this model could explain the increased number of *ato-*expressing cells in discs mutant for *sca*. In simulating cluster formation in *sca*-depleted discs, we noted that the initial effect of *sca* depletion, observed at the very first columns, is very different from that observed at later stages. At first, depletion of *sca* led to smaller clusters, as expected from the deletion of an activator. Subsequently, however, the lack of an activator led to noise amplification, resulting in ‘catastrophes' of large elongated clusters. At the second stage of refinement, lateral inhibition often failed, leading to the frequent formation of twinning, as reported experimentally^13^ (Fig. 4e). This failure to refine was explained by the slower kinetics of Delta accumulation, reflecting the increased inhibition of *ato* in the absence of its activator Sca. This slower kinetics of Delta increased the error frequency of the lateral inhibition process^23^. Finally, the reported genetic interactions between Sca and Delta-Notch signalling, and between Sca and Egfr signalling^14^,^18^,^24^ are similarly explained by this model (Supplementary Note 8 and Supplementary Fig. 4).

### Evidence that Sca functions as an ato activator

**Figure 5:**
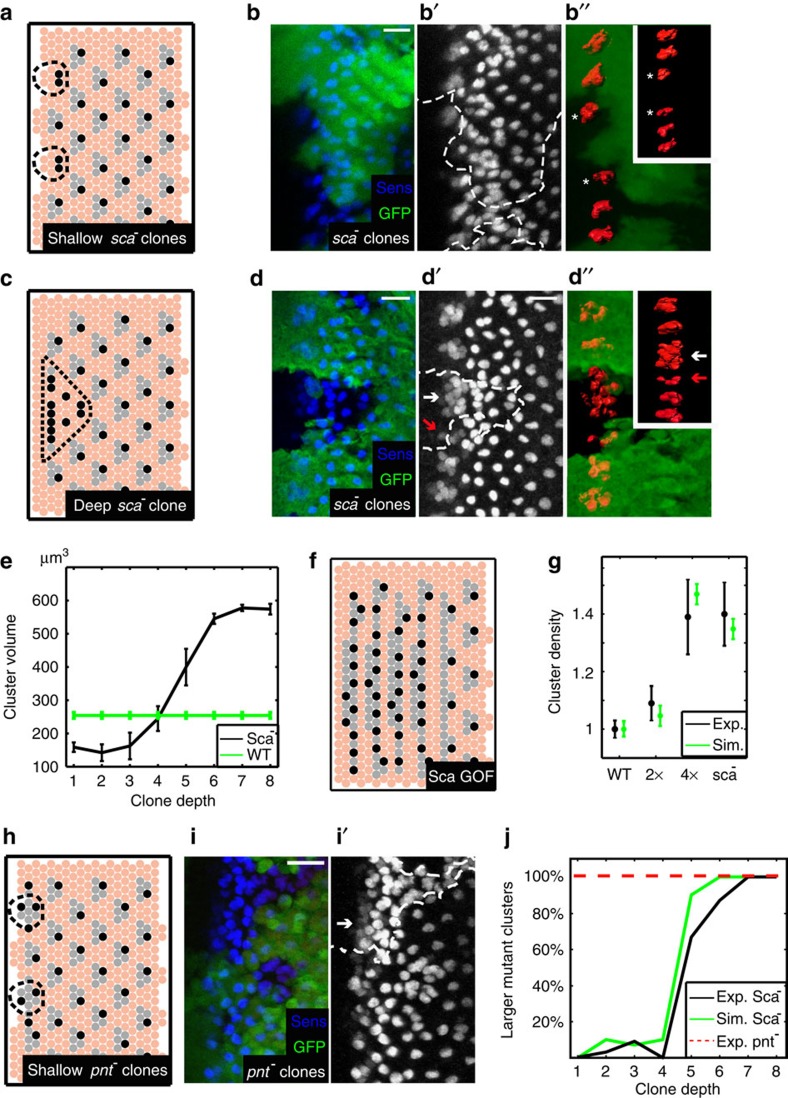
Clonal analysis supports Sca role as activator of *ato* expression. (**a**) Simulations of shallow clones (enclosed by broken line) result in smaller clusters. In grey are cluster cells that were inhibited by the selected cells in each cluster (in black). (**b**) The nuclei stained for Sens are in blue, and shallow clones that lack *sca* are visualized by lack of GFP signal in green indicated by the white broken line. In the right panel (**b′′**), clusters are marked in 3D in red using the Imaris imaging software that quantifies the volume of each cluster. White asterisks indicate smaller clusters inside the clones. Between the indicated clusters there is a gap where no cluster has formed. Inset shows the clusters after 90° rotation. Scale bar, 10 μm. (**c**,**d**) Same as **a**,**b**, but for a deep clone. White arrows in **d** indicate a larger cluster, while red arrows indicate a smaller cluster in the shallow region of the clone. Scale bar, 10 μm. (**e**) Shown are the average cluster sizes within *sca* mutant clones as a function of clone depth. Clusters larger than 600 μm^3^ where not included. Number of clones analysed are *n*=55,13,10,6,4,4,4,4 for clones of depth one to eight columns, respectively. Error bars denote s.e. (**f**) Simulation of *Sca gain-of-function* (GOF) discs. (**g**) Cluster density in *sca−/−* mutants and in *sca gain-of-function* flies carrying two or four (2 × and 4 ×) copies of *roE-sca* (an enhancer fragment of the rough gene that drives sca expression) was quantified in ref. 25 (black bars). Regions containing 17 or 18 clusters in at least four discs from each genotype were subjected to this quantitative analysis. Cluster density in our simulations was quantified by dividing the number of cells in the clusters by the number of total cells in 72 simulations for each genotype. Error bars denote s.e. (**h**,**i**) Same as **a**,**b** for clones depleted of *pnt.* (**j**) The percentage of larger *sca−/−* and *pnt−/−* mutant clusters as a function of clone depth. Number of *sca−/−* clones analysed is the same as in **e**. At least three *pnt−/−* clones were analysed for each depth.

The increased instances of ‘catastrophes' and of twinning in *sca-*depleted discs is therefore consistent with our predicted function of Sca as a diffusible activator, and result from error propagation and limited capacity for subsequent refinement. We next wished to test Sca function more directly by examining its initial function observed before error propagation. We reasoned that generating clones of *sca-*depleted cells would enable focusing on the initial role of Sca in cluster formation, and further distinguish between immediate effects and those that are due to error propagation. Specifically, clones positioned only at the furrow (the region of cluster formation) will report directly on cluster size and spacing, while deep clones, which extend from the furrow into the posterior patterned regions, will report on error propagation. To examine the expected effects, we first simulated such clones. Indeed, shallow clones (less than four cells deep) resulted in small clusters (Fig. 5a).

To experimentally test these predictions, we generated mutant *sca* clones in eye discs of third instar larvae, stained these discs for the nuclear protein Sens and imaged them using confocal microscopy. To measure cluster size, we needed to count the number of stained nuclei. Since nuclei are positioned at different Z-positions within the tissue, we used a specialized programme (Imaris) that integrated data from different *z* planes and enabled identifying all stained nuclei (Supplementary Note 9).

We considered only clones positioned at the MF, and measured the number of rows that extend into the posterior differentiated region (clone depth). We then averaged the cluster volumes at different depths. Indeed, clusters situated in shallow clones abutting the MF were smaller compared with their neighbouring well-aligned clusters situated in WT regions (Fig. 5b and red arrows in Fig. 5d). On the other hand, clusters situated in deeper clones spanning several columns behind the MF were larger, resembling the catastrophes observed in our simulations (Fig. 5c,d). To monitor the critical *sca−/−* clone depth from which noise accumulates to a sufficient amount, and in which instead of smaller clusters (compared with WT clusters) catastrophes start to appear, we quantified cluster volume as a function of clone depth (Fig. 5e). Our quantification indicates that the critical clone depth is approximately four columns of cells, in agreement with our simulations.

An additional test for Sca function is the phenotype of discs that overexpress Sca. In our simulations, overexpression of an activator leads to increased selection of cells at the furrow but does not slow the kinetics of Delta accumulation in selected cells (Fig. 5f). This is consistent with the phenotype reported in a previous study showing an increased cluster density at the furrow, similar to the phenotype observed in the *sca*-depleted discs, but no instances of ‘twinning' (Fig. 5g)^25^. To calculate cluster density in our simulations, we divided the number of cluster cells by the total number of cells, and set the average WT density to be 1. Our simulations give rise to similar cluster densities in the different mutant backgrounds to those reported (green bars in Fig. 5g).

Together, these results are consistent with Sca acting as an activator, rather than an inhibitor of *ato* production.

### Egfr signalling generates an inhibitor of ato expression

If Sca functions as an activator of *ato,* Egfr should trigger the main diffusible inhibitor. This role of Egfr, however, was debated in a study reporting that the reduced cluster spacing observed in *Egfr* mutants is not due to impaired patterning but to increased apoptosis^26^. To more directly examine the effect of the predicted Egfr-dependent inhibitor, while avoiding broader consequences of disturbing Egfr signalling (such as Hid-dependent cell death), we examined eye discs mutated for *pointed (pnt)*, the transcription factor mediating Egfr-dependent transcriptional responses^27^,^28^. Indeed, extensive convergence of clusters, representing recruitment of excess cells, was observed (Supplementary Note 10 and Supplementary Fig. 5). Conversely, when Egfr signalling was increased by introducing one copy of the *Egfr* gain-of-function allele *Ellipse* (*Elp*), cluster size was reduced^29^,^30^ (Supplementary Fig. 5e). Quantitatively, we find that cluster size within clones is always larger than that of WT, irrespective of clone depth, as expected from the deletion of an inhibitor (Fig. 5h–j). These results support the implicated inhibitory role of Egfr during cluster formation.

## Discussion

The *Drosophila* eye imaginal disc is patterned through a template-based lateral inhibition process, in which differentiation proceeds as a propagating wave^31^. A similar mechanism is found in other differentiating systems, including feather formation in the chick^32^, and cone cell induction in zebrafish^33^. By examining the sensitivity of this mechanism to spatial heterogeneity in biochemical parameters, we show that template-based lateral inhibition fails to generate reliable patterns. We predicted that this circuit should contain a short-range diffusible activator whose action is critical for reducing noise sensitivity. Through a combination of theory and experiments, we re-interpreted the phenotypes of a main player in this system, the Sca protein, and show that it functions as an activator, rather than an inhibitor of cluster formation. By reassigning Sca, this novel function of our model reconciles biochemical evidence showing that Sca negatively regulates Notch (the main *ato* inhibitor)^15^,^22^, with the observed increase in *ato-*expressing cells in eye discs depleted of *sca*^13^,^24^. We show that a patterning system that combines propagating lateral inhibition with a short-range activator is capable of withstanding considerable noise. Other mechanisms may function in parallel to reduce sensitivity to noise in this system (for example, cell constriction in the MF, see Supplementary Note 11 and Supplementary Fig. 6).

In general, the need to buffer genetic and environmental perturbations dramatically restricts the possible designs of patterning networks^34^. We have shown here that buffering spatial heterogeneities confines the mechanisms that generate periodic patterns through a dynamic, template-based lateral inhibition. Underlying this increased sensitivity is the difficulty of coordinating inhibitory patterns generated from adjacent sources. It may therefore be interesting to study its applicability to related mechanisms employing periodic patterning; in particular to those generating self-organized patterns through Turing-like instabilities that were shown to be sensitive to spatial heterogeneity and stochasticity^35^,^36^. Further studies are required to examine whether rapidly acting diffusible activators may also increase robustness of those related modules.

## Methods

### Fly strains and clonal analysis

The following lines were used for mutant clone generation: *ey-flp; FRT82B Ubi-GFP* (obtained from the Bloomington Stock Center), *FRT82B pntΔ88* (obtained from Helen McNeill, Samuel Lunenfeld Research Institute, Ontario, Canada), *FRT42D sca*^*BP2*^ and FRT42D *ubi-GFP* (obtained from Bloomington stock #7320). Homozygous mutant clones for *sca*^−^ and *pnt*^*−*^ were generated by FRT-mediated recombination using *ey*-flp. *sca*^−^ clones were generated in *ey-Flp*; FRT42D *sca*^*BP2*^/FRT42D *ubi-GFP* third-instar larvae. *pnt*^−^ clones were generated using *ey-Flp*; *FRT82B pntΔ88/FRT82B Ubi-GFP*. Dissection was carried out following a further 48-h incubation at 25 °C. *pnt* eye-specific knockdowns were induced by crossing *ey3.5-Gal4* flies (obtained from the Bloomington Stock Center) to *UAS-pnt RNAi* (VDRC ID KK105390) flies.

### Immunohistochemistry

Standard fixation and staining protocols were used on dissected third instar larva eye imaginal discs. Briefly, after dissection on ice-cold PBS, fixation using 4% paraformaldehyde was performed. Washes and permeabilization were carried out using 0.1% Triton X-100. Blocking was then performed for 30 min using bovine serum albumin (0.1%). Primary antibodies used for incubation overnight were anti-Sens (guinea pig 1:2,000, obtained from H. Bellen), anti-FasIII (mouse monoclonal 1:20, Developmental Studies Hybridoma Bank), anti-Dlg (mouse monoclonal 1:100, Developmental Studies Hybridoma Bank), anti-GFP (chick 1:2,000, Aves Labs) and anti-Dcp-1 (rabbit 1:100, Cell Signaling Technology). Secondary antibodies used for 2-h incubation were anti-guinea pig Alexa 647 (1:800), anti-mouse Alexa 488 (1:800), anti-rabbit Alexa 488 (1:800), anti-mouse Alexa 555 (1:800) and anti-chick Dy-Light (1:800), all obtained from Molecular Probes.

### Quantification

Quantification of cluster size within and outside *sca*^−^ clones and of the distances between clusters was obtained using the Imaris imaging processing software.

### Numerical approach and parameters

Equations were solved by a custom-written Matlab programme implementing an explicit forward Euler method. See Supplementary Methods for detailed simulations.

The equations for the extended network described in Fig. 4 describe the full selection dynamics: starting with the formation of clusters and continuing with their refinement to a single selected cell. These equations are discussed in length in Supplementary Note 7. The equation for *h* is the same as in Figs 1 and 3 and is given fully by Supplementary equation (12.3). The remaining equations are:


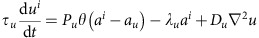



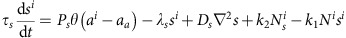



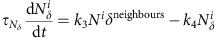



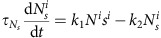



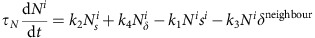



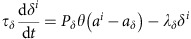



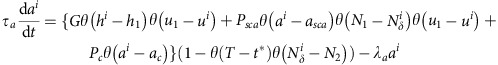


*u* is the short-range inhibitor and *s* is the short-range activator, which now acts as a Notch inhibitor; *N* and *δ* represent free (unbound) Notch and Delta; *N*_*s*_ represents the complex of *N* with *s* and *N*_*δ*_ represents the complex of *N* with *δ*; *a* is the cell-autonomous activator. The typical timescale, production rate and degradation rate of each component are denoted by *τ*, *P* and *λ*, respectively. *D*_*u*_ is the diffusion coefficient of *u*.

The equation for the diffusible activator *s* now includes also the rate by which it binds and unbinds Notch (*k*_1_ and *k*_2_, respectively). Notch binds and unbinds its ligand *δ* from neighbouring cells (denoted as *δ*^neighbours^) at rates *k*_3_ and *k*_4_, respectively, to form the activated complex *N*_*δ*_.

## Additional information

**How to cite this article:** Gavish, A. *et al.* Periodic patterning of the *Drosophila* eye is stabilized by the diffusible activator Scabrous. *Nat. Commun.* 7:10461 doi: 10.1038/ncomms10461 (2016).

## Supplementary Material

Supplementary Information Supplementary Figures 1-6, Supplementary Tables 1-3, Supplementary Notes 1-11 Supplementary Methods and Supplementary References.
