# Supplementary material for: Periodic patterning of the Drosophila eye is stabilized by the diffusible activator Scabrous
Source: Nat Commun. 2016 Feb 15;7:10461. doi: 10.1038/ncomms10461 (PMC4756378; doi:10.1038/ncomms10461)
Supplement: Supplementary Information — Supplementary Figures 1-6, Supplementary Tables 1-3, Supplementary Notes 1-11 Supplementary Methods and Supplementary References. [file ncomms10461-s1.pdf]

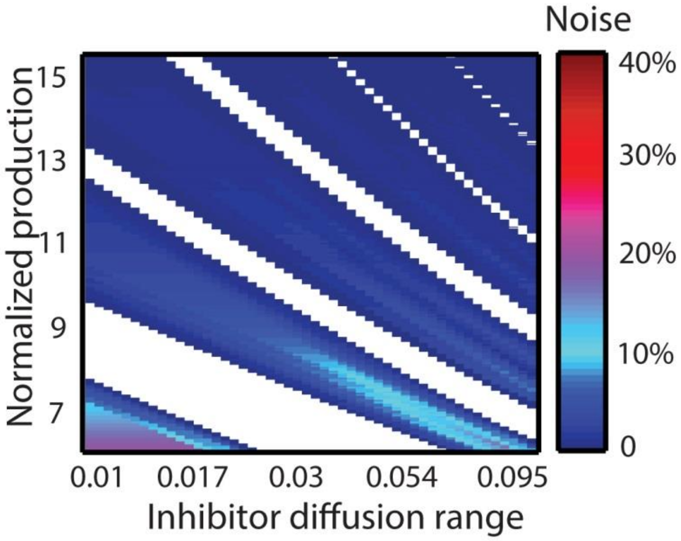

Supplementary Figure 1 (related to Figure 1). Phase diagrams for adding noise to all parameters

Shows same phase diagram as Figure 1d, for when adding noise to all parameters in the system except parameters that determine *h* propagation. The diagrams are practically identical, differing only in the maximal noise that is permitted before patterns deteriorate.

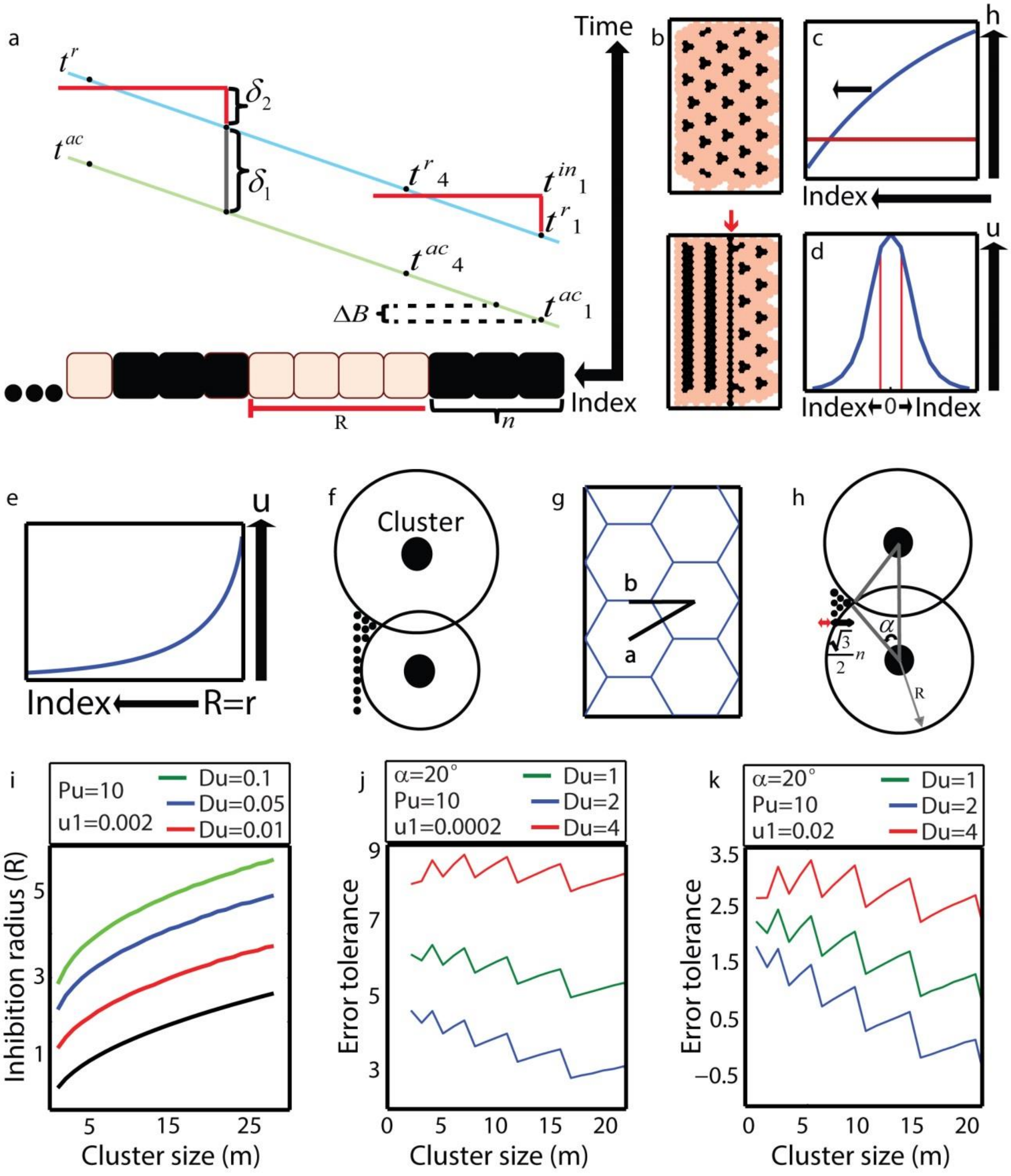

Supplementary Figure 2 (related to Figure 2). One and two dimensional lateral-inhibition based models

(a) Pattern formation in one dimension. Each pattern is characterized by two parameters: Cluster size n, and the distance between clusters, R. Time is on the horizontal axis and cell position on the vertical axis. The number of cells that become refractory before the first cell in the cluster produces inhibition (red vertical line) determines cluster size. t_i^r is the time when a cell becomes refractory to inhibition, relative to the time of activation. t_1^{in} is the time when the first cell produces inhibition. δ_1 is given in Eq. (2.5).

(b) Line template propagates as a line. Upper plane, simulation of cluster formation corresponding to plane 1 in figure 3c. Lower plane, as a result of a line induced at the position of the red arrow, the pattern continues to propagate as stripes.(c) h profile at a given time as described in Eq. (12.3). The red line indicates some threshold h_1 above which a cell is activated. h propagates as a wave in the direction of the black arrow. Thus, different cells sense h_1 at different times. (d) u profile of a one-dimensional cluster as described in Eq. (2.8). u decays exponentially out of the cluster boundaries (indicated by red lines). (e) u profile outside of a two-dimensional cluster as described in Eq. (3.9) in a sagittal plane. u decays exponentially away from the cluster edge $R = r$. (f) Catastrophe occurrence in the presence of noise. Minimal perturbations in the inhibition radiuses result in the selection of too many cells. (g) Distance between neighboring cells and columns. The distance between centers of neighboring cells is set to be 1, and the distance between columns b is derived accordingly. (h) Definition of the $Error\ tolerance$. The $Error\ tolerance$ is the distance from the cluster edge to the closest column ahead that is not restricted by inhibitory radiuses (red arrow). The larger the $Error\ tolerance$, the less chances for a catastrophe to occur as a result of fluctuations in the inhibition radius. R is the inhibition radius and n is the number of columns. (i) Inhibition radius R as a function of cluster size m for different diffusion constants. The black profile is the radius of the cluster and its dependence on cluster size is shown in Note 4. The smaller the cluster, the more sensitive is R to fluctuations in m, as indicated by an increased incline for smaller clusters.(j-k**)** Maximizing the $Error\ tolerance$. $Error\ tolerance$ as a function of cluster size for several parameter sets. The lower threshold u_1 (j) resultes in a larger span between the $Error\ tolerance$ profiles for the different diffusions. In both cases, the red curves describe parameter sets for which the maximal $Error\ tolerance$ is obtained for multi-cellular clusters of six cells. The reason for the tooth-like shape of the curves is that the maximum $Error\ tolerance$ for a given column is obtained when the column if full. Once a column starts filling, adding more cells to that column will only increase the inhibition radius and thus contribute to the $Error\ tolerance$.

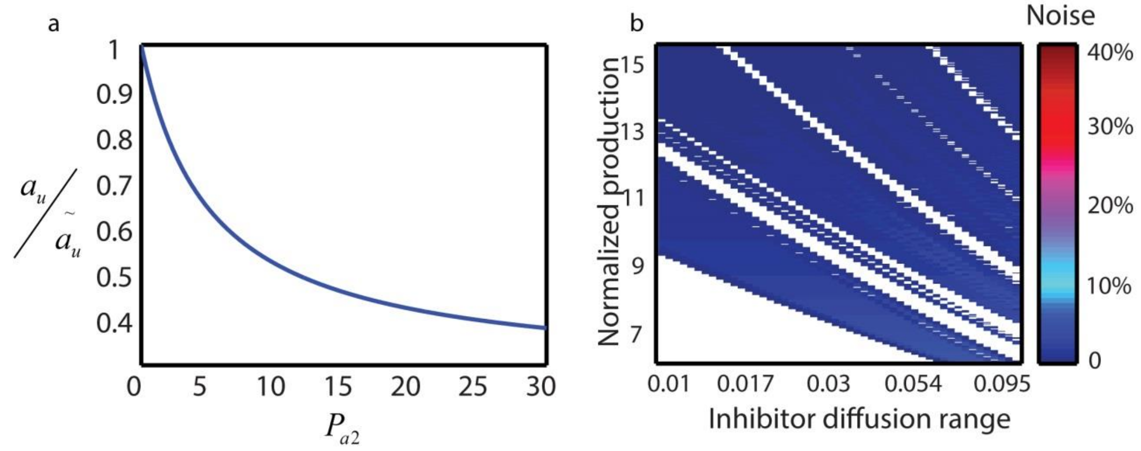

Supplementary Figure 3 (related to Figure 3). Delayed auto activation vs. diffusible activator.

(a) Delayed auto-activation does not provide new functional properties. Solving Eq. (5.4) for the parameters in Supplementary Table 1 with $a_d = 0.8$ ($a_a < a_d < a_u$) and with P_{a2} varying from zero to 30, demonstrates that regarding the dynamics of cluster formation, addition of delayed auto-activation is equivalent to an effective decrease in the threshold for inhibition production and therefore does not contribute to robustness. (b) Same as Supplementary Figure 1 for the diagram in Fig. 3c.

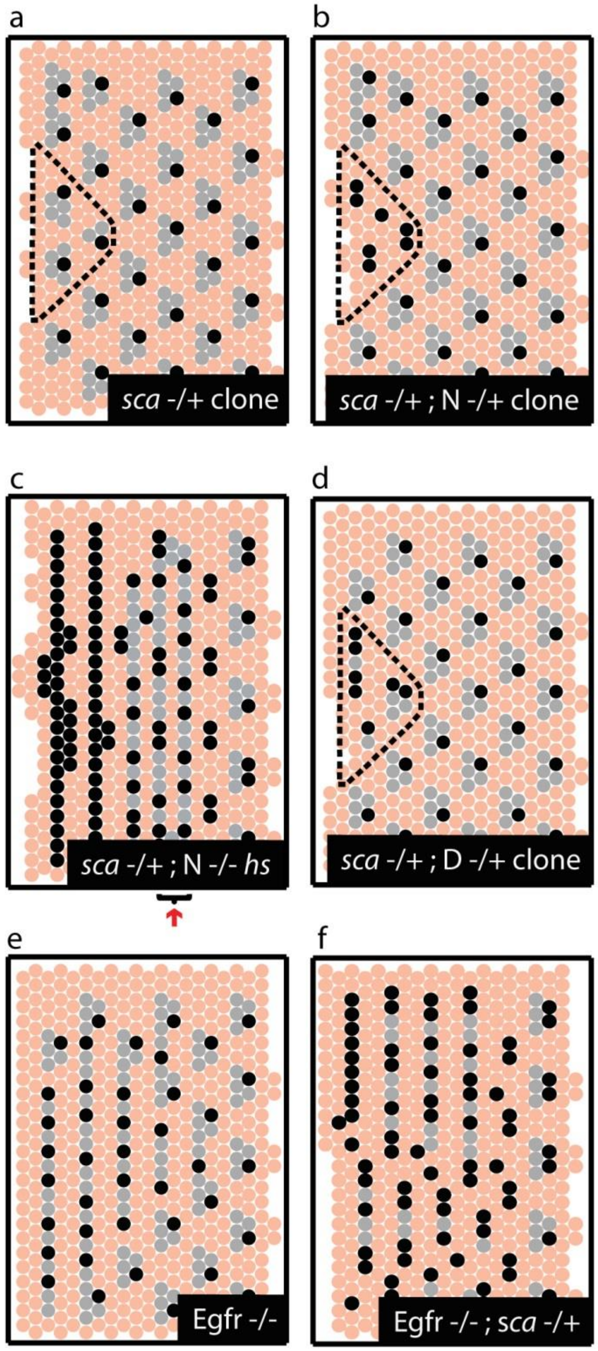

Supplementary Figure 4 (related to Figure 4). Sca interactions with Delta, Notch and Egfr

(a) Sca heterozygote. Sca production was set to 50% within the region indicated by the broken line. Patterning was not severely affected. (b) *sca*/Delta double heterozygote. Sca and Delta production were set to 50% within the region indicated by the broken line. Because of impaired lateral inhibition, an adjacent pattern of twining occurred that later will lead to eye roughening. (c) Induction of stripes. As previously described, temporarily

abolishing Notch signaling (indicated by the red arrow) in a Sca mutant result in the formation of a propagating pattern of stripes. (d) $sca/$Notch double heterozygote. Sca and Notch production were set to 50% within the region indicated by the broken line. Because of impaired lateral inhibition, twining occurred that later will lead to eye roughening. (e) Patterning in $Egfr^{-}$ discs. Production of u was set to 30% and catastrophes started to appear from column 13 due to the insufficient inhibition range. (f) $Egfr/sca$ double mutant. Production of s together with production of u was set to 30% and catastrophes appeared more rapidly, from column 9.

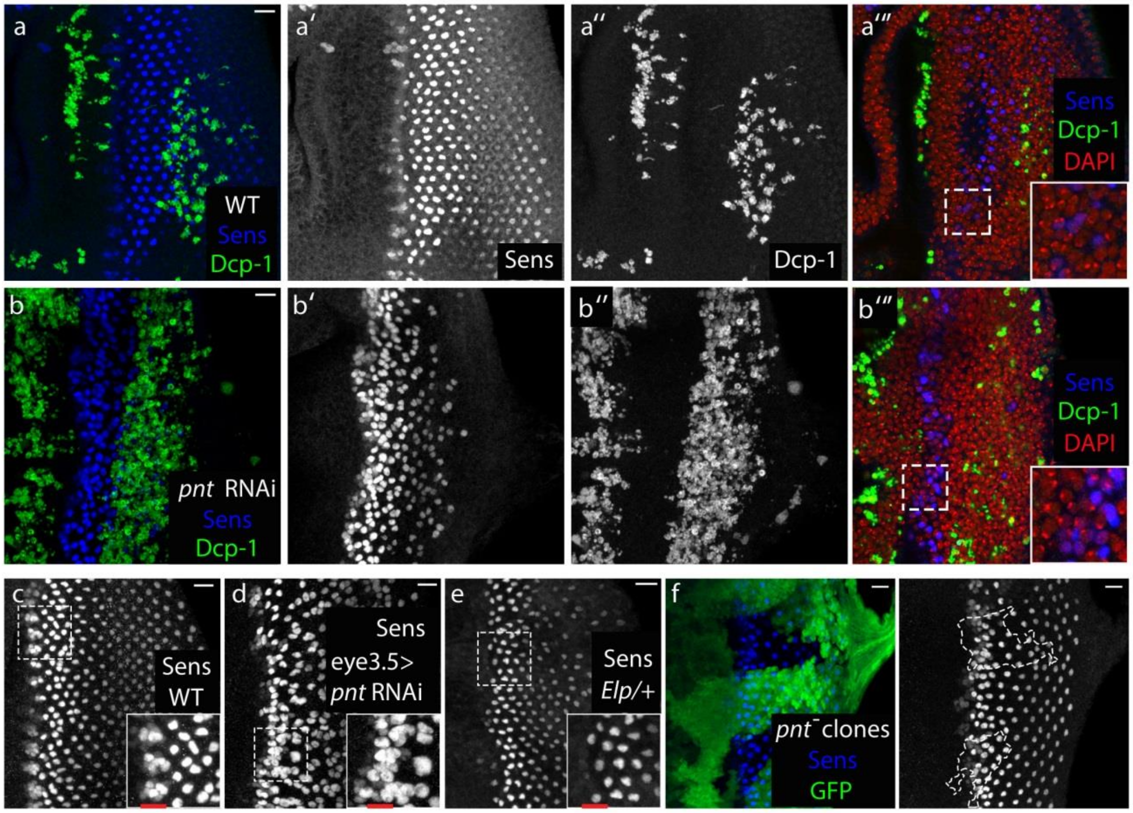

Supplementary Figure 5 (related to Figure 6). *sca* and *Egfr* mutations
(a-b) *Egfr* phenotype is not a result of increased apoptosis at the MF. Sens is in blue; the apoptosis marker (Dcp-1) is in green; nuclei are marked by DAPI in red in the right most panels (the insets represent a magnified view of the boxed regions in the MF). (a) Wild-type eye disc where little apoptosis is seen anterior and posterior to the MF. (b) *pnt* knockdown background where more apoptosis is seen anterior and posterior to the MF, but none is seen within the furrow. The boxed regions show a normal morphology of the nuclei with a similar density compared to WT. (c-e) Egfr acts as an inhibitor to limit cluster size. The insets represent a magnified view of the boxed regions, overlapping and immediately posterior to the MF (red line). (c) In WT discs, Sens is expressed in evenly spaced clusters within the furrow, which resolve posteriorly into individual Sens-expressing photoreceptors. (d) Knockdown of the Egfr mediator *pnt* results in extensive convergence of clusters, confirming that Egfr plays a role in the initial cluster spacing. (e) Elevated basal activity of the Egfr pathway results in a decrease in cluster size. *Ellipse* is a dominant, gain-of-function mutation in the *Egfr* gene, thought to cause over-activation of Egfr signaling. In eye discs heterozygous for this allele, smaller Sens clusters or single cells are seen within the furrow, consistent with higher basal levels of inhibitor. (f) *pnt* mutant clones. Sens is in blue and *pnt* mutant clone is visualized by lack of GFP signal in green (also marked by broken white lines). An extensive convergence of clusters is seen within the clones, in comparison to the normal formation of clusters outside the clones.

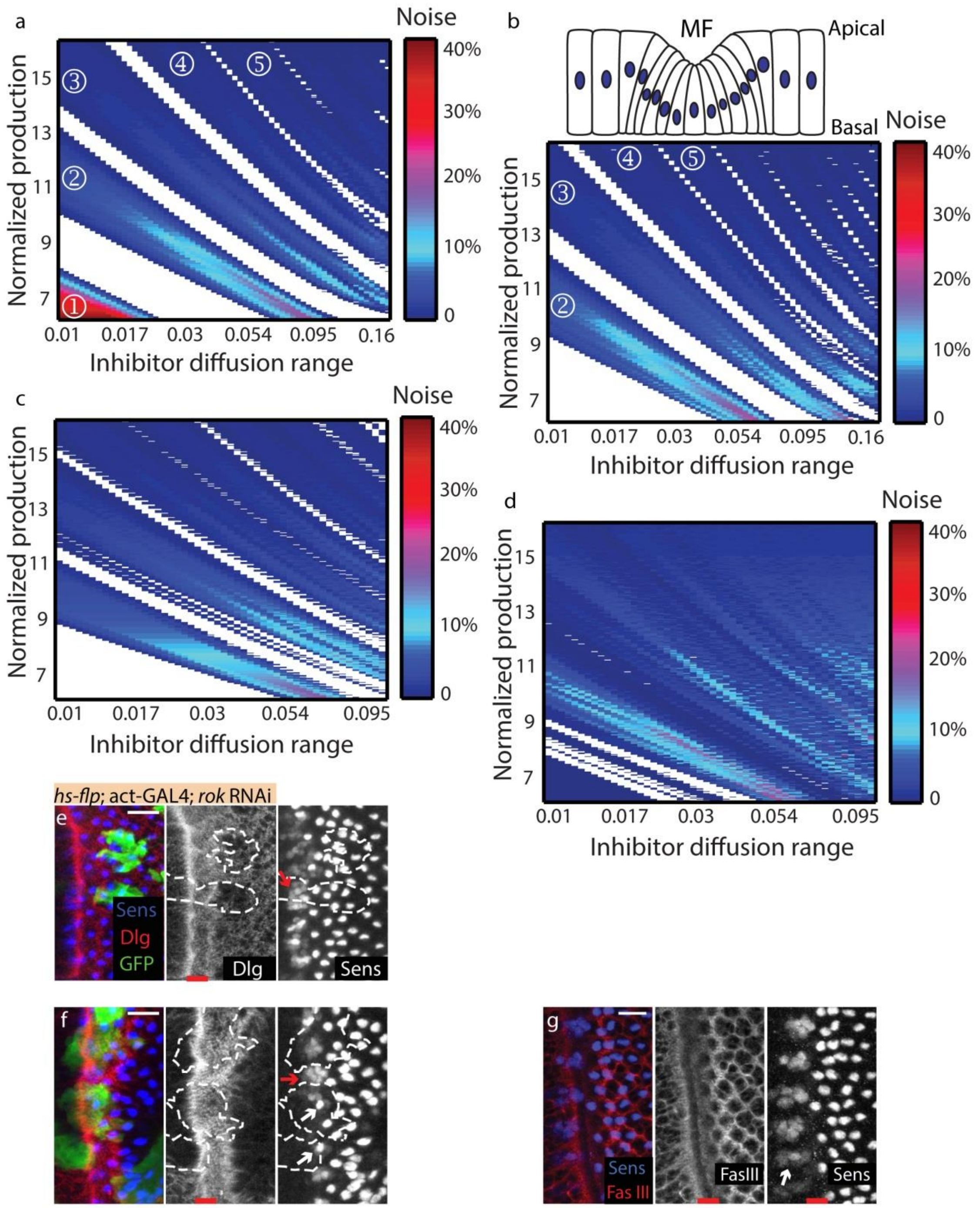

Supplementary Figure 6. Cell constriction in the furrow improves robustness. (a-d) Phase diagrams. (a) Same as figure 1d, where the inhibitor diffusion range was extended a little further. (b) Apical constriction in the furrow. Tissue indentation which accompanies cluster formation within the MF may function to effectively increase cell proximity and thereby increase robustness. After taking constriction into account, more

noise could be added to patterns that fit the biological setting in the *Drosophila* eye shown in (a). See Supplementary Note 12 for numerical approach and parameters. (c-d) same as (a-b) for the phase diagram in figure 3c, where (c) is without accounting for constriction and (d) is after accounting for constriction. Significantly more parameter sets allowed patterning, and more noise could be added. (e) *rok* RNAi-induced clones (visualized by GFP signal in green). Sens (blue) marks nuclei of differentiating cells and Dlg (red) marks the basolateral cell membrane. Interfering with cell constriction resulted in formation of large clusters within the furrow (red arrow). (f) In some cases, reduced constriction resulted in formation of single differentiated cells instead of clusters (white arrows). Red arrow indicates a normally sized WT cluster between clones. These different phenotypes are consistent with the presence of a short-range activator, where the phenotype depends on when constriction was perturbed relative to cluster formation. (g) Cluster formation is not always synchronized with the furrow. Cell outlines are visualized with FasIII (red), and differentiating cells with the nuclear differentiation marker Sens (blue). The furrow is marked by the red line. White arrow points at a cluster that formed anterior to the furrow. As furrow-associated constriction extends anterior to the MF, it may still facilitate robustness when cluster formation also precedes the furrow. Scale bars: 10 μm

$\tau_a = 1.2$	$P_a = 5$	$\lambda_a = 1$	$D_h = 600$	$a_a = 0.4$	$h_1 = 1$
$\tau_h = 100$	$P_h = 2.4$	$\lambda_h = 1$	$D_u = [0.01,0.1]$	$a_u = 2.9$	u_1=2·$[10^{-2}, 10^{-6}]$
$\tau_u = 0.015$	$P_u = 10$	$\lambda_u = 1$	$G = 0.9$	$v = 0.25$	

Supplementary Table 1.
Parameters for Fig. 1d, 1e and 3e.

$P_a = 3$	$P_s = 10$	$S = 2.5$
$v = 0.75$	$\lambda_s = 1$	$D_s = 0.016$
$a_u = 1.55$	$\tau_s = 0.01$	$s_1 = 0.01$

Supplementary Table 2.
Parameters for Fig. 3e.

$\tau_a = 1.5$	$P_{sca} = 4$	$a_c = 0.4$	$v = 1.15$	$\lambda_\delta = 1$	$a_\delta = 1.2$
$P_c = 3$	$a_{sca} = 0.01$	$T = 6.5$	$k_1 = 5$	$k_3 = 0.85$	
$a_u = 1.2$	$N_1 = 0.03$	$N_2 = 1$	$k_2 = 1$	$k_4 = 4.1$	
$\tau_{N_\delta} = 0.05$	τ_{N_s}=0.05	$\tau_N = 0.05$	$\tau_\delta = 0.05$	$P_\delta = 20$	

Supplementary Table 3.
Parameters for Fig. 4c.

Supplementary Note 1: A dynamic template-based lateral-inhibition model for cluster formation: model formulation and numerical analysis

Model formulation:

In this section, we formulate the dynamic template-based lateral inhibition model described in the text, and analyze its behavior in one dimension. This model was presented in previous work and was studied analytically by M. W. Pennington & D. K. Lubensky [1]. Some of the derivations in this section repeat this earlier work, and are brought here for completeness.

The model considers three variables: a long-range, non-autonomous activator h, a non-autonomous short-range inhibitor u, and a self-inducing activator a. A selected cell is a cell that stably expresses a. We are searching for a solution whereby at the end of the dynamics, selected cells are found in clusters of size n, organized in a hexagonal lattice. This dynamics captures the first part of the selection dynamics – the formation of clusters. These clusters are subsequently refined into a single selected cell through further action of the Notch-Delta lateral inhibition circuit. We consider the complete dynamics in Note 7 below. However, since proper selection of single cells is critically dependent on the robust formation of the cluster, we focus our analysis here on cluster formation. We also note that in the special case $n = 1$, discussed by Lubensky and colleagues, a single selected cell is obtained already at the first part of the dynamics.

The initiation of a expression in a particular cell requires that the level of the activator h in this cell's position exceeds some threshold h_1.Once expression begins, a accumulates in a cell-autonomous manner. When its levels exceed some threshold a_a, it begins inducing its own expression, and in effect becomes refractory to possible inhibition by the inhibitor u produced by adjacent cells. Subsequently, when its level exceeds a second (higher) threshold a_u it begins producing the inhibitor u, which is secreted and rapidly diffuses to inhibit a production in neighboring cells. Cells are inhibited only if they haven't become refractory prior to that point. Finally, when a accumulates above a third threshold a_h it begins producing h. This long-range activator is secreted and diffuses to initiate a expression in non-inhibited cells. The differential equations describing this dynamics are given by:

(1.1)

$$\tau_a \frac{da^i}{dt} = P_a\theta(a^i - a_a) - \lambda_a a^i + G\theta(h^i - h_1)\left(1 - \theta(u^i - u_1)\right)$$

$$\tau_u \frac{du^i}{dt} = P_u\theta(a^i - a_u) - \lambda_u a^i + D_u \frac{\partial^2 u}{\partial x^2}$$

Where τ_a, τ_u denote the typical time scales characterizing the dynamics of a and u, respectively; λ_a, λ_u are the respective degradation rates and are used to normalize all other parameters; P_a, P_u are the respective production rates; D_u is the diffusion constant of u. $\theta(a)$ is the Heaviside step function, which is equal to 1 for positive values and zero otherwise. The upper-script indexes (i) indicate the grid site and $\frac{\partial^2 A}{\partial x^2} = A_{i+1} + A_{i-1} - 2A_i$ is the grid one-dimensional Laplacian operator. The system in Eq. (1.1) needs to be complemented with boundary conditions. Without loss of generality, we assume

reflecting boundary conditions. In addition, initial conditions need to be specified. We begin the differentiation from a pre-pattern of selected cells at the posterior-most region. h is therefore expressed initially at the posterior region and diffuses to anterior regions where it triggers a expression. As more anterior cells are selected to express a, those cells begin expressing h, leading to the progression of the pattern. For simulation purposes, we use an analytical approximation for h (formulated in Note 12, Eq. 12.3), by which h is a uniformly propagating wave with speed v.

Supplementary Note 2: Analytical approximation

The numerical analysis had shown that only a small subset of parameters result in a periodic pattern. To better understand how the different parameters control the eventual pattern, we attempted also an analytical approximation for the continuous form of Eq. (1.1).

A precise analytical solution of the dynamics defined by our model equations (1.1) is not possible. To approximate the temporal dynamics, we consider the following observations. First, we note that all cells are equivalent and differ only in their spatial position. This position impinges on the dynamics by setting the time at which a particular cell becomes exposed to h levels that are sufficiently high to initiate a expression (providing that the cell was not yet inhibited). We denote this activation time of a cell in a position i along the anterior-posterior axis as t_i^{ac}. The difference in this activation time between neighboring cells is defined by the velocity by which h propagates along the tissue with speed v, which we assume to be constant in time (Eq. 12.1).

Second, we note that once a expression is initiated in a given cell, it accumulates and subsequently reaches different thresholds, first the threshold for refractoriness and then the threshold for producing the inhibitor u. We can summarize this dynamics by the time it takes the cell to reach those thresholds. Thus, we consider two additional times (a) the time lag from activation of cell i until it becomes refractory and maintains a expression, even if it is subjected to inhibition, which we denote as t_i^r. Note that this time is independent of relative cell position in the cluster. (b) The time lag from the activation of the cell i until it begins expressing the inhibitor u, which we denote as t_i^{in}. Note that this time depends on the position of the cell within the cluster. We assume that once the inhibitor is expressed in the first cell, it quickly diffuses and inhibits the selection of all cells that haven't become refractory yet. (Note that u will also have some effect on the refractory cells by reducing the rate of a accumulation. Consequently, those refractory cells will reach the inhibitory threshold at different times depending on their position in the cluster. Since those times are relatively fast compared to h propagation, positive-dependent effect does not influence the selection dynamics).

The formation of a cluster can now be understood through the interplay between the times above, as is illustrated schematically in Supplementary Figure 2a. The key two parameters we need to define are the size of the cluster n, and the distance between adjacent clusters, R. The spacing between clusters, R, is determined by the distance over which the inhibitor is effective. Cluster size is determined by the number of cells that become refractory before being repressed by the inhibitory signal generated by the first cell in the cluster. It is therefore given simply by:

(2.1)

$$n = \left\lceil \frac{t_1^{in} - t_1^r}{\Delta t^{ac}} \right\rceil$$

Where $\Delta t^{ac} = \frac{1}{v}$ is the time gap between activation of two neighboring cells, and is defined by the speed of h propagation. t_1^r and t_1^{in} are the respective times when the first cell in the cluster becomes refractory and produces inhibition.

In order to complete this analytical approximation, we need to write the different times specified above, as well as the distance between clusters, R, in terms of the molecular parameters specified in the set of equations Eq. (1.1) above. This is what we do in the following section.

The relationship between the different timing and the model's molecular parameters

Consider a cell i positioned at a distance x along the anterior-posterior axis. This cell will be activated at time t_i^{ac} defined by the condition $h(x, t_i^{ac}) = h_1$.

Once activated, this cell will start producing a according to Eq. (1.1), whose solution when adopting its continuous form, is:

(2.2)

$$a_i(t) = \left(-\frac{G}{\lambda_a}\right) e^{-\frac{\lambda_a}{\tau_a}(t - t_i^{ac})} + \frac{G}{\lambda_a}$$

The cell will become refractory when $a_i(t_i^{ac} + t_i^r) = a_a$; hence according to Eq. (2.2):

(2.3)

$$t^r = \left(\frac{\tau_a}{\lambda_a}\right) \ln[\frac{G}{G - a_a \lambda_a}]$$

Note that we defined the refractory time relative to the time of activation. After becoming refractory, a production increases (see Eq. (1.1)). Hence, its levels are now given by:

(2.4)

$$a_i(t) = \left(a_a - \frac{G + P_a}{\lambda_a}\right) e^{-\frac{\lambda_a}{\tau_a}(t - (t^r + t_i^{ac}))} + \frac{G + P_a}{\lambda_a}$$

For the first cell in the cluster, Eq. (2.4) holds from the time the cell became refractory until the time it began producing inhibition. For the other cells in the cluster, Eq. (2.4) holds until they are inhibited by the inhibitor secreted by the first cell, which will occur when $a_i\left(t_i^{ac} + t^{in}\right) = a_u$:

(2.5)

$$t^{in} = t^r + \delta_1 \quad ; \quad \delta_1 = \left(\frac{\tau_a}{\lambda_a}\right) \ln[\frac{a_a \lambda_a - (G + P_a)}{a_u \lambda_a - (G + P_a)}]$$

Cluster size

As mentioned above, a cell will belong to a cluster if it became refractory before the first cell in the cluster started to produce inhibition. Accordingly, the cluster size n is determined by:

(2.6)

$$n\frac{1}{v} + t^{r} < t_{1}^{in}$$

Here, $n\frac{1}{v}$ is the time from the activation of the first cell in the cluster until the activation of the n'th cell, which depends on the rate of h propagation. Substituting Eq. 2.3, 2.5 in Eq. 2.6 we obtain:

(2.7)

$$n = \left\lceil\frac{v\tau_{a}}{\lambda_{a}}\right\rceil \ln[\frac{a_{a}\lambda_{a} - (G + P_{a})}{a_{u}\lambda_{a} - (G + P_{a})}]$$

Therefore, cluster size n is determined by the time difference between refractoriness and inhibition-production within the first cell, and the velocity of h propagation v. This result will be important to later understand the cause of catastrophes in two-dimensions. Note that for cluster size $n > 1$, changes in furrow velocity greatly impact on cluster size. As furrow velocity was shown to change by ~3-folds during patterning [2], this may be a limitation of the inhibitor only model. Importantly, addition of an activator (see below), alleviates this dependency.

The distance between clusters

In order to estimate the distance between clusters R, we next find the steady-state distribution of the inhibitor u. We assume separation of time-scales, so that a and u respond rapidly to the slowly propagating h. Accordingly, after a cluster is formed, h will continue to propagate and will induce activation only in cells which are out of the inhibition range. The first cell that is not inhibited by u will be the first cell in the new cluster. Thus, we are interested in finding the effective range of inhibition around an isolated cluster of a given size n.

Consider a cluster of length $2L$, where $2L = nK_{z}$, K_{z} being the typical cell size. We can solve u based on the continuous form of Eq. (1.1), where z position is measured relative to the cluster center. Due to our choice of scaling, by which each cell has a diameter of 1 (see Note 4), we can substitute $n = 2L$ and obtain (Supplementary Figure 2d):

(2.8)

$$u(z) = \begin{cases} \frac{P_u}{\lambda_u}\left(1 - e^{-\sqrt{\frac{\lambda_u}{D_u}}\frac{n}{2}}\cosh\left(\sqrt{\frac{\lambda_u}{D_u}}z\right)\right) & |z| < \frac{n}{2} \\ \frac{P_u}{\lambda_u}e^{-\sqrt{\frac{\lambda_u}{D_u}}|z|}\sinh\left(\sqrt{\frac{\lambda_u}{D_u}}\frac{n}{2}\right) & |z| \geq \frac{n}{2} \end{cases}$$

Where z is measured relative to the center of the cluster. According to Eq. (2.8), cells subjected to high enough u above the threshold $u \geq u_1$ will be inhibited, defining the inhibitory range to be:

(2.9)

$$\mathrm{R} = \sqrt{\frac{D_u}{\lambda_u}}\ln[\frac{P_u}{\lambda_u u_1}\sinh\left(\sqrt{\frac{\lambda_u}{D_u}}\frac{n}{2}\right)]$$

Condition for successful recruitment:

A necessary condition for our model to work is that the inhibition produced by the first cell will be effective in stopping cluster growth. Effectively, this requires that the cluster size n, will be smaller than the radius of the inhibition produced by the first cell. This gives us the following condition:

(2.10)

$$\mathrm{R}_1 > n$$

Where R_1 is the inhibition radius produces by one cell. Substituting Eqs. (2.7) and (2.9) into Eq. (2.10) gives the essential constraint on the speed of h propagation, v.

Supplementary Note 3: A dynamic template-based lateral-inhibition model for cluster formation: Two-dimensional analysis

In this section, we describe the unique properties of the model in two dimensions, as compared to the one-dimensional case described above. The major differences are that first, the diffusion is now conducted in two dimensions and second, the cluster size n derived above now describes the number of columns, and not the number of cells.

Qualitatively, the solution for h is not affected, as it propagates with line symmetry. Accordingly, the terms describing the different times (activation time, refractory time and inhibitory time) remain unchanged, but now apply for all cells in a given column. In contrast, the steady-state solution for u is somewhat different as it now describes an inhibition circle around each cluster, rather than a one-dimensional exponential decay. Accordingly, the distance between clusters, R, now has to be recalculated.

We consider again the continuous steady-state condition for the inhibitor levels $\frac{du}{dt} = 0$:

(3.1)
$$\tau_u \frac{du}{dt} = P_u\theta(a - a_u) - \lambda_u a + D_u \nabla^2 u = 0$$

Changing variables, Eq. (3.1) can be written as:

(3.2)
$$\lambda^2 \nabla^2 u(x, u) - u(x, y) + \eta(x, y) = 0 \;\; ; \;\; \lambda^2 = D_u \;\; ; \;\; \eta(x, y) = P_u\theta(a - a_u)$$

For solving this equation, we assume a spherical-symmetrical cluster and use the Green formalism:

(3.3)
$$\lambda^2 \nabla^2 G(\vec{R}) - G(\vec{R}) + \delta(\vec{R} - R_0) = 0$$

By solving this equation first in Fourier space, and transforming back to real space, we obtain:

(3.4)
$$G(\vec{R}) = (\frac{1}{2\pi})^2 \int_0^\infty \frac{k_R}{\lambda^2 {k_R}^2 + 1} dk_R \int_0^{2\pi} e^{ik_R \vec{R} \cos\theta} d\theta$$

Using the Jacobi-Anger expansion, we obtain:

(3.5)
$$\int_0^{2\pi} e^{iz\cos\theta} d\theta = 2\pi J_0(z) + 2\sum_{n=1}^{\infty} \frac{i^n}{n} J(z) \sin(n\theta) \Big|_0^{2\pi} = 2\pi J_0(z)$$

Where $J_0(z)$ is the Bessel function. Eq. (3.4) and (3.5) give:

(3.6)
$$G(\vec{R}) = (\frac{1}{2\pi})^2 \int_0^\infty \frac{k_R}{\lambda^2 {k_R}^2 + 1} J_0(k_R \vec{R}) dk_R = \frac{1}{2\pi} \frac{1}{\lambda^2} K_0(\frac{\vec{R}}{\lambda})$$

Where $K_0(z)$ is the modified Bessel function of the second kind.
From this, the solution for the distribution of u is given by:

(3.7)
$$u(\vec{R}) = \int d^2 R_0 G(\vec{R} - R_0)\, \eta(\vec{R} - R_0)$$

Where $|\vec{R}| < r$ is the domain of the cluster in which cells produce inhibition. Eq. (3.6) and (3.7) give:

(3.8)

$$u(\vec{R}) = \int_0^{\infty} R_0 dR_0 \int_0^{2\pi} d\theta \frac{1}{2\pi}\frac{1}{\lambda^2} K_0\left(\frac{\vec{R}-R_0}{\lambda}\right) P_u \theta(r - R_0)$$
$$= \frac{P_u}{D_u}\int_0^{r} R_0 K_0\left(\frac{\vec{R}-R_0}{\lambda}\right) dR_0$$

Denoting $\frac{P_u}{D_u} = \alpha[\frac{mol}{m^2}]$ the steady state solution for u is:

(3.9)

$$u(\vec{R}) = \frac{\alpha}{2}\vec{R}\left\{\pi R L_{-1}\left(\frac{\vec{R}}{\lambda}\right) K_0\left(\frac{\vec{R}}{\lambda}\right) + \left(\pi R L_0\left(\frac{\vec{R}}{\lambda}\right) + 2\lambda\right) K_1\left(\frac{\vec{R}}{\lambda}\right)\right\}$$
$$-\frac{\alpha}{2}(\vec{R} - r)\left\{\pi \vec{R} L_{-1}\left(\frac{\vec{R}-r}{\lambda}\right) K_0\left(\frac{\vec{R}-r}{\lambda}\right) + \left(\pi \vec{R} L_0\left(\frac{\vec{R}-r}{\lambda}\right) + 2\lambda\right) K_1\left(\frac{\vec{R}-r}{\lambda}\right)\right\}$$

Where r is the radius of the cluster, $\vec{R}$ is where u is calculated and L_{-1}, L_0 are the modified Struve functions. The inhibition radius R is then obtained numerically by demanding $u(\vec{R} = \mathrm{R}) = u_1$ (Supplementary Figure 2e).

Supplementary Note 4: Definition of the 'Error tolerance' and its dependence on cluster size

In two dimensions, most of the errors of the pattern are due to 'catastrophes', namely instances where a line-like pattern of selected cells is observed (Supplementary Figure 2f). Such catastrophes occur due to fluctuations in inhibition radiuses, which result in spatial regions that are not properly inhibited (Figure 2b). Let us define the $Error\ tolerance$ as the distance from the cluster edge (anterior-most column selected) to the closest column that is not restricted by inhibitory radiuses. The larger the $Error\ tolerance$, the smaller is the probability that fluctuations in the inhibition radiuses will result in catastrophes. A zero or negative $Error\ tolerance$ means that a column selected is not restricted by inhibition radiuses and therefore causes a catastrophe. The parameters that determine the $Error\ tolerance$ are the cluster size, n, the inhibition radius R, and the point at which the inhibition circles intersect. The $Error\ tolerance$ decreases with increasing cluster size, but increases with increasing the inhibitory radius. Scaling the diameter of each cell to be 1, and using the fact that the distance between adjacent columns is $\frac{\sqrt{3}}{2}$ (Supplementary Figure 2g), and the radius of a cluster of m cells is $\frac{\sqrt{m}}{2}$, the $Error\ tolerance$ is then given by (Supplementary Figure 2h):

(4.1)

$$Error\ tolerance = \mathrm{R} - \mathrm{R}\sin(\alpha) - n\frac{\sqrt{3}}{2}$$

Notably, changing the mean value of cluster size and inhibitor radius (n and R) affect not only the $Error\ tolerance$, but also their sensitivity to spatial variations. For example, increasing n would have a dual function: on the one hand, as noted by Eq. (4.1), it will have a direct effect on reducing the error tolerance. Yet on the other hand, it will increase the inhibitory radius, and further decrease its sensitivity to fluctuations (Supplementary Figure 2i), and in this way would increase the $Error\ tolerance$. Therefore, analytical solutions for parameters that minimize the $Error\ tolerance$ are difficult, and we have searched for such parameters numerically (Supplementary Figure 2j and 2k). Nevertheless, even for optimized parameter sets that minimize the $Error\ tolerance$ catastrophes still occur frequently, indicating on an inherent sensitivity to noise in the dynamic lateral-inhibition model.

Supplementary Note 5: addition of delayed intracellular auto-activation does not improve robustness:

As an attempt to improve the robustness of the model, we examined a model that includes a localized feedback that functions at a delay. Such a feedback was included in the previous model by Lubesky and colleague [1], to capture the effect of Senseless (Sens), a transcription factor whose expression is induced by *ato* and can in turn feedback to activate *ato.* We note that the model discussed above already includes a localized positive feedback, captured by the auto-activation of *ato* itself. We still examined whether the Sens-based feedback, which functions at a delay and introduces additional parameters to the system, could provide some robustness. Note that this cell-autonomous feedback is very different from the Sca-based positive feedback we introduce below, which is secreted and induces activation in near-by cells.

To simulate the function of Sens (denoted by *d*) we repeated our simulations of the model (Eqs. (1.1)) including also a delayed auto-activator whose dynamics is defined by

(5.1)

$$\tau_d \frac{dd^i}{dt} = P_d\theta\left(a^i - a_d\right) - \lambda_d d^i$$

$$\tau_a \frac{da^i}{dt} = P_a\theta\left(a^i - a_a\right) + P_{a2}\theta\left(d^i - d_d\right) - \lambda_a a^i + G\theta\left(h^i - h_1\right)\left(1 - \theta\left(u^i - u_1\right)\right)$$

Where τ_d is the typical time-scale of d; P_d is the production rate of d; λ_d is the degradation rate of d. When d reaches the threshold d_d, a is further produced in the rate P_{a2}. The equations for h and u and are the same as in Eq. (1.1). We assume that the threshold for auto-activation by a is lower than the threshold for delayed auto-activation by d (a_a< a_d. Otherwise auto activation by d wouldn't be delayed as it would precede that by a). Under this assumption, there are two possible scenarios. If the threshold for delayed auto-activation is higher than the threshold for inhibition production (a_u< a_d),

the dynamics of cluster formation is determined exclusively by auto-activation by a (see Note 2). Therefore, in this case delayed auto activation does not affect the dynamics of cluster formation and cannot contribute to robustness. The second option is that the threshold for delayed auto-activation is lower than the threshold for inhibition production ($a_a < a_d < a_u$). A simple mathematical calculation shown here demonstrates that this option is equivalent to an effective reduction in the threshold for inhibition production a_u in a system lacking this component. Therefore, adding a cell-autonomous positive feedback does not provide the system with any new functional capacities and in particular cannot enhance robustness.

Following Eqs. (2.3) and (2.5), we denote the time when a cell initiates delayed auto-activation as t_i^d given by:

(5.2)

$$t^d = t^r + \delta_2 \quad ; \quad \delta_2 = \left(\frac{\tau_a}{\lambda_a}\right) \ln\left[\frac{a_a\lambda_a - (G + P_a)}{a_d\lambda_a - (G + P_a)}\right]$$

A cell will start producing the inhibitory signal at:

(5.3)

$$t^{in} = t^d + \delta_3 \quad ; \quad \delta_3 = \left(\frac{\tau_a}{\lambda_a}\right) \ln\left[\frac{a_d\lambda_a - (G + P_a + P_{a2})}{a_u\lambda_a - (G + P_a + P_{a2})}\right]$$

Let us now denote the threshold for inhibition production and the time of inhibition production in the previous system, lacking delayed auto-activation, as $\widetilde{a_u}$ and $\widetilde{t^{in}}$ respectively. The times when inhibition is produced in both systems are the same when (we demand $t_i^{in} = \widetilde{t_i^{in}}$ and insert Eqs. (2.5) and (5.3)):

(5.4)

$$\frac{\widetilde{a_u}\lambda_a - (G + P_a)}{a_u\lambda_a - (G + P_a + P_{a2})} = \frac{a_d\lambda_a - (G + P_a)}{a_d\lambda_a - (G + P_a + P_{a2})}$$

Supplementary Figure 3a shows the ratio $\frac{a_u}{\widetilde{a_u}}$ after solving Eq. (5.4) demonstrates that addition of delayed auto-activation is equivalent to an effective decrease in the threshold for inhibition production and therefore does not provide new functional properties.

Supplementary Note 6: Adding a diffusible activator to the "dynamic template-based lateral-inhibition model"

In this section, we describe our extended model, including a diffusible activator. As we show in the main text, this model is significantly more robust to spatial variations in the model's parameters, and in particular rescues most catastrophes. The motivation for adding an activator was in fact to allow the formation of large clusters that are stable against the catastrophes, as we described (Figure 3c). Increased cluster size provides

further robustness due to decreased fluctuations in inhibition radiuses, as more cells participate in inhibition production.

We assume a diffusible activator s whose expression is induced when a reaches some threshold a_s. We assumed that this threshold is the same as the threshold for refractoriness a_a and is therefore lower than the threshold at which the inhibitor u is produced.

The equations describing this extended model are:

(6.1)

$$\tau_a \frac{da^i}{dt} = P_a\theta(a^i - a_a) - \lambda_a a^i + G\theta(h^i - h_1)\left(1 - \theta(u^i - u_1)\right) + S\theta(s^i - s_1)\left(1 - \theta(u^i - u_1)\right)$$

$$\tau_u \frac{du^i}{dt} = P_u\theta(a^i - a_u) - \lambda_u a^i + D_u\nabla^2 u$$

$$\tau_s \frac{ds^i}{dt} = P_s\theta(a^i - a_a) - \lambda_s a^i + D_s\nabla^2 s$$

$\tau_s, P_s, \lambda_s, D_s$ are the respective time-scale, production rate, degradation and diffusion constants of the short-range activator. All other parameters are the same as in the original model in Eq. (1.1).

Note that since s is secreted and diffuses to nearby cells, and since its production is rapid, at a relative low level of a, its expression propagates to several column (through a activation). This propagation stops only once the inhibitor u is produced by the first cell to be selected in the cluster. In effect, the rapid propagation of s now replaces the slower propagation of h in determining cluster size. The key difference between propagation of h and the propagation of s is their symmetry: while h propagates in a line-symmetry, and is therefore susceptible to catastrophes, s propagates with a radial symmetry and therefore is not susceptible to catastrophes. The radial symmetry in which s propagates allows clusters to partially not be circumscribed by inhibitory radiuses from behind (with a negative $Error\ tolerance$ that would otherwise result in catastrophe), therefore enabling the formation of larger clusters, with reduced sensitivity of the inhibitory radius to production and diffusion rates, while avoiding the occurrence of catastrophes.

More specifically, cluster size will now be given by:

(6.2)

$$n = \left\lceil \frac{t_1^{in} - t_1^{r}}{\Delta t_s^{ac}} \right\rceil$$

Where $\Delta t_s^{ac} = \frac{1}{v_s}$ is the time gap between activation of two neighboring cells, given now by v_s, the effective velocity by which s expression propagates between two neighboring cells (replacing v_h used in the core model, c.f. Eq. (2.1) above). v_s depends on the molecular parameters, and can be approximated analytically, assuming that s

diffuses rapidly and effects neighboring cells practically immediately upon its secretion. Δt_s^{ac} is therefore approximated by the time it takes a to reach the threshold for s production and is calculated as described in Note 1 above (c.f. Eq. (2.2)),

(6.3)

$$\Delta t_s^{ac} = \frac{1}{v_s} \approx \left(\frac{\tau_a}{\lambda_a}\right) \ln[\frac{G}{G - a_a \lambda_a}]$$

The only note is that this time now differs somewhat between cells due to our assumption that cells which receive both activating signals $\boldsymbol{s}$ and $\boldsymbol{h}$ produce a faster than cells which receive only the $\boldsymbol{h}$ or $\boldsymbol{s}$ activation signal.

Supplementary Note 7: Simulating the full dynamics of the system: from undifferentiated cells, through cluster formation, to the selection of single cells

Our main work so far focused on the formation of the pre-clusters expressing *ato*, as improper selection of those clusters necessary result in the loss of the final pattern. Those clusters are subsequently refined to generate a single selected cell. In this section, we extend our simulations to show that a single set of equations can describe the full selection dynamics: starting with the formation of clusters and continuing with their refinement to a single selected cell. To this end, we extended our model in three ways:

1. We explicitly consider the function of the Notch-Delta pathway in our model. During cluster formation and their refinement, this is the primary pathway mediating lateral inhibition: The Delta ligand is activated by the selected *ato*-expressing cells and, in turn, binds Notch in nearby cells thereby repressing *ato* expression in those neighboring cells.
2. Including the Notch pathway enables us to more precisely model the function of Scabrous. Biochemical evidence has shown that Scabrous binds to Notch and inhibits its activity [3,4]. Therefore, rather than assuming that Sca directly activates *ato* expression, we include this ability of Sca to inhibit Notch activity.
3. We continue the simulations for a longer time, simulating not only the rapid stages of cluster formation, but also their refinement by the Notch-Delta pathway until a single cell is selected.

The full network is described schematically in Figure 4, with the dynamics of the different components defined by the system of reaction-diffusions equations:

(7.1)

$$\tau_u \frac{du^i}{dt} = P_u \theta\left(a^i - a_u\right) - \lambda_u a^i + D_u \nabla^2 u$$

$$\tau_s \frac{ds^i}{dt} = P_s \theta\left(a^i - a_a\right) - \lambda_s s^i + D_s \nabla^2 s + k_2 {N_s}^i - k_1 N^i s^i$$

$$\tau_{N_\delta}\frac{d{N_\delta}^i}{dt} = k_3 N^i \delta^{neighbors} - k_4 {N_\delta}^i$$

$$\tau_{N_s}\frac{d{N_s}^i}{dt} = k_1 N^i s^i - k_2 {N_s}^i$$

$$\tau_N \frac{dN^i}{dt} = k_2 {N_s}^i + k_4 {N_\delta}^i - k_1 N^i s^i - k_3 N^i \delta^{neighbor}$$

$$\tau_\delta \frac{d\delta^i}{dt} = P_\delta \theta(a^i - a_\delta) - \lambda_\delta \delta^i$$

$$\tau_a \frac{da^i}{dt} = \{G\theta(h^i - h_1)\theta(u_1 - u^i) + P_{sca}\theta(a^i - a_{sca})\,\theta(N_1 - {N_\delta}^i)\theta(u_1 - u^i) + P_c\theta(a^i - a_c)\}(1 - \theta(T - t^*)\theta({N_\delta}^i - N_2)) - \lambda_a a^i$$

Here the equations for long-range activator h and for the diffusible inhibitor u are the same as in Eq. (1.1). The equation for the diffusible activator s now includes also the rate by which it binds and unbinds Notch (k_1 and k_2, respectively, with N_s representing the complex of N with s). Notch binds and unbinds its ligand δ from neighboring cells (denoted as $\delta^{neighbors}$) at rates k_3 and k_4 respectively to from the activated complex N_δ. The typical time scales of N_δ, N_s, N and δ are captured by $\tau_{N_\delta}, \tau_{N_s}, \tau_{N,} \tau_\delta$, respectively.

The most notable difference of this model with respect to the core model discussed above is the dynamics of a. This dynamics now depends explicitly on the level of a, u and N_δ , with h inducing a activity, while both u and N_δ repressing a expression. Roughly, the dynamics of a can be described as follows. As in the core model, a begins to be produced once h level increase above some threshold h_1 and it begins to induce its own expression therefore not requiring the h signal when exceeding some threshold a_{sca}. The cells can still be inhibited by either u or activated Notch (N_δ) if exceeding the respective thresholds specified, until a reaches a second threshold a_c. When exceeding this threshold, a becomes refractory to inhibition by u and is considered as part of the pre-neuronal cluster.

Notably, at this stage, a expression can still be inhibited by activated Notch, although now higher levels of activated Notch are required for this inhibition and therefore its ligand Delta produced by *ato* expressing cells needs to exceed higher levels. Once one of the cells produces sufficient Delta, it inhibits all of its neighboring cells thereby remaining the only selected cell. Finally, following the observation that lateral-inhibition processes are confined to a certain time window [5], we assume that a cell that is not inhibited within a finite time window is selected by default. This is equivalent to assuming an additional a threshold which renders it refractory to Notch inhibition.

Scabrous acts as an effective activator of *ato* expression in this model by out-competing Delta, and therefore reducing the level of the activated Notch (N_δ). More specifically, since Notch is used also at stages prior to cluster formation [6,7] we assume that some Notch-Delta complex are present even at the beginning of the dynamics and can therefore rapidly reach the first threshold N_1, inhibiting *ato* auto-production through

the second term of the RHS. Sca reduces this N_δ level, therefore facilitating *ato* expression.

Using numerical simulations, we find that this extended model can indeed capture the full dynamics, including cluster formations and their refinement (Figure 4b). Further, this model shows the same (relative) robustness to spatial variations, as found for our simplified model including the diffusible activator (Figure 4c and 4d). We note that for the parameters used here (see Supplementary Table 3) cluster refinement may allow oscillations of N_δ levels, as observed in about 10% of the cases.

Supplementary Note 8: Explaining the phenotypes of the different Sca mutants

The extended model described above, enables us to examine whether the model captures the phenotype of different mutations and perturbations previously described to effect R8 selection. Of particular interest are the phenotypes observed when perturbing Scabrous. We attribute Scabrous the role of an activator of *ato* expression, contrasting the prevailing notion that Scabrous functions as an inhibitor of *ato* expression.

Scabrous loss of function (LOF) at the MF

Biochemical evidence has shown that Sca binds Notch to inhibit its function. Since Notch is an inhibitor of *ato* expression during cluster formation and refinement, this would imply an activator role for Sca. Still, Sca was attributed a role in inhibiting *ato* based on the phenotypes of discs depleted of *sca* that show an increased number of R8 cells, which often occur in two adjacent neighboring cells. Such 'twinning' is never observed in wild-type discs. While this phenotype indeed seems to indicate that Sca inhibits *ato* expression, we reasoned that this eventual phenotype might be more difficult to interpret due to the dynamic aspect of the patterning. For example, if the lack of an activator promotes catastrophes, resolution of those catastrophes may be more difficult by the subsequent lateral inhibition resulting in twinning. Further, since Sca continues to inhibit Notch also during the second, refinement stage, it may slow the dynamics which could also result in twinning.

To examine rigorously whether our model can explain the formation of twinning upon *sca* depletion, we simulated the full model Eqs. (7.1) assuming *sca* is deleted. We followed the full dynamics, including cluster formation and refinement. Simulation results are shown as before, using the same parameters used to simulate the wild-type disc (Figures 4 and Supplementary Figure 4).

As predicted for a system lacking an activator, deletion of *sca* at the stage of cluster formation results in the formation of catastrophes (Figure 4e). Clusters are first formed closer to each other, and subsequently catastrophes are observed. Further, the refinement of the larger cluster is not precise and twinning is often observed, similar to the results observed experimentally. Examining the simulations more closely, we noted that the lateral inhibition process becomes significantly slower, which impairs cluster refinement, as the inhibition process does not culminate within the available time

window. The restriction of the clusters to single R8 cells was indeed shown to take longer in sca^{-} discs, in consistency with our model [8]. Lack of sca slows down the process of cluster refinement, due to the loss of auto-activation.

Scabrous interaction with Notch and Delta signaling

Sca interacts genetically also with Notch and Delta, an interaction that was also taken as evidence for its function as an inhibitor of ato expression (rather than an activator, as we suggest). Specifically, reducing the levels of either Notch or Delta dominantly enhance the phenotype of sca heterozygotes by significantly increasing the number of ato expressing cells [8]. Since the Notch-Delta pathway acts as a primary inhibitor of ato expression during cluster formation and refinement, this genetic interaction appears to implicate Sca also as an inhibitor. However, as noted above, the eventual phenotype is difficult to interpret. The biochemical evidence that Sca inhibits, rather than activates Notch, further questions the interpretation of the genetic interaction between Sca and Delta/Notch.

To examine whether our model explains the phenotype, we simulated Eqs. (7.1) while assuming two-folds reduced production of Sca and both Sca and Delta (Supplementary Figure 4a and 4b). As can be seen, our model, in which Sca functions as an effective activator of $ato,$ captures the reported genetic interaction. Thus, while heterozygotes to either Sca or Delta show no significant change in patterning, the double heterozygotes shows a pronounced increase in the frequency of adjacent R8 selected cells. This increase in the frequency of 'twinning' reflects the reduced efficiency of the lateral inhibition process. The positive genetic interaction between Delta/Notch and Sca, despite their opposite effect, is explained by the dynamic nature of the patterning: As noted above, removing sca results in slower dynamics, leading to a slower accumulation of Delta. Reduced levels of Delta further slows down the dynamics, and in occasional cases differences between adjacent cells are too small to be resolved by the lateral inhibition within the time window provided, leading to the selection of two adjacent cells.

An additional phenotype was described when sca heterozygote was combined with transient inhibition of Notch using the Notch temperature-sensitive allele. Following this inhibition, ato was expressed in a stripe-like manner, and this stripe pattern was maintained for several subsequent differentiating columns (Supplementary Figure 4c). This, again, was successfully explained by our model: transient inhibition of Notch allowed high ato expression in a full line, and this perturbation propagated further due to the changes in initial conditions. Once an initial column of stripes is induced, instead of a column of evenly spaced clusters, the pattern will continue to propagate as stripes due to the template-based mode of pattern propagation (Supplementary Figure 2b). Further, transient propagation of this line is facilitated by the slower lateral-inhibition dynamics caused by sca heterozygosity. Simulations of clones in which both Sca and Notch production were reduced by two-fold also result in twining due to a cumulative effect of slower Delta kinetics and impaired lateral-inhibition (Supplementary Figure 4d).

Scabrous gain of function (GOF) at the MF

Overexpression of Sca was tested in previous works. A work by Ellis and coworkers [9] analyzed ommatidial spacing after over-expression of Sca in the morphogenetic furrow using the *ro*-enhancer. They observed irregularly spaced clusters in the MF and a higher density of differentiated R8 cells .Therefore, the GOF phenotype is similar to the LOF phenotype, in the sense that both lead to the selection of a larger number of *ato* expressing cells. Notably, following cluster refinement the phenotype differs, as twining is not seen upon Sca overexpression.

To examine whether our model explains the phenotype, we simulated Eq. 7.1 assuming a two-fold and four-fold increase in Sca production. Indeed, at the stage of cluster formation, our simulation results predict the formation of merged cluster, consistent with the experimental observation (Figure 5f). Further, despite being large, refinement of these clusters is mostly successful resulting in additional R8 cells, but no twinning, again consistent with the experimentally observed phenotype (Figure 5f). We note that the absence of twinning reflects the faster dynamics of the Notch-dependent lateral inhibition in the mutant over-expression Sca.

Scabrous interaction with Egfr signaling

An additional finding that supported the notion that Sca functions as an inhibitor of *ato* expression is its positive genetic interaction with Egfr signaling, which functions as an inhibitor of *ato* expression (Note 10). Thus, previous work has shown that when both Egfr signaling and *sca* are removed, more R8s are formed and closely spaced columns of cells can be observed [10]. Therefore, impairing both signals enhances the two individual phenotypes observed when either signal is impaired individually. This would imply that Sca and Egfr signaling have a similar (rather than an opposite) role regarding *ato* expression. However, also here, the eventual phenotype is difficult to interpret as the system is dynamical and errors that arise in the first stage of cluster formation may propagate to cause different phenotypes at the refinement stage. For example, if the lack of the activator would result in the formation of catastrophes, its removal would indeed cause additional R8 selection, as we have shown above.

To examine whether our model explains the phenotype, we simulated Eq. (7.1) once after reducing production of u and then after reducing production of both u and s (Supplementary Figure 4e and 4f). The latter cases resulted in a more severe phenotype in which catastrophes occurred faster and resulted in twining. The quicker destruction is due to the even higher necessity for an activator when the inhibition radiuses are small, as in such cases cells are unconfined by inhibition more readily. As previously explained in Note 6, the radially propagating activator can allow clusters to partialy not be circumsribed by inhibition without resulting in catastrophes. Therefore, the presence of a short-range activator can somewhat alleviate the impared inhibition, whereas its absence can result in a more severe phenotype.

Scabrous over-expression in front of the MF

Cells that are in front of the MF have not yet begun the processes of cluster formation and refinement. Therefore, their different phenotypes are not explained by our model, which is concerned only with the processes of cluster formation and refinement. It should be noted that the functional role of Notch in that pre-differentiation stage is very different, and in fact opposite to its role in inhibiting *ato* expression during cluster formation. Thus, the 12-15 cell rows in front of the furrow show a very weak, uniform activation of *ato*. Rather than inhibiting this expression, Notch is now required for *ato* expression as it inhibits the primary inhibitors of *ato* at this stage Hairy and EMC [7]. Accordingly, the role of Sca, as a Notch inhibitor, at this pre-differentiated stage should also be different from its role during cluster formation and refinement: an inhibitor of *ato* expression during this pre-differentiation stage and an activator during cluster formation and refinement. This explains the observation that ectopically expressing Sca in front of the MF results in the rapid loss of *ato,* and was in fact used to verify the function of Sca as a Notch inhibitor [3]. We emphasize again, however, that since the role of Notch changes from the pre-differentiation to the differentiation stage, this phenotype is not relevant for Sca functions at the timing of cluster formation and refinement.

The predicted effect of Scabrous loss of function (LOF) clones behind the MF

As explained above, our model predicted two distinct phenotypes of sca^{-} clones, depending on their size. For small clones that fall in the furrow, we expect to see smaller clusters. This is because if Sca is an activator, its deletion at the stage of cluster formation will result in smaller clusters. In contrast, for large clones that extend into the differentiated region we expect to see a propagation of errors (including the formation of catastrophes) that are characteristics of a system lacking a short-range activator. Therefore, in those clones we expect to see larger and fused clusters.

This is indeed what we observe. Figure 5a shows a simulation of small clones, spanning only upon the MF, not allowing noise to propagate from behind. Figure 5c shows a large clone of sca^{-} that spans several columns of cells behind the MF, allowing noise to propagate and excess cells to be chosen. In figures 5e and 5j we show a quantification of this phenotype, and show that critical clone depth for noise to propagate and catastrophes to start occurring is ~4 columns. This analysis provides more proof that at the stage of cluster formation Sca is an activator.

Supplementary Note 9: Quantification of the sca^{-} clone phenotype

Here we explain in more detail how we quantified cluster sizes in the $\boldsymbol{sca^{-}}$ clones. As is explained above, the predicted phenotype of the $\boldsymbol{sca^{-}}$ clones depends on their size. Deletion of *sca* in large regions, that span several columns behind the MF, enables the propagation of errors and formation of excess selection of cell (catastrophes)

that are characteristics of a system lacking a short-range activator. In contrast, in small clones that do not span much behind the MF, the observed phenotype is due merely to the primary role of Sca, and is not a consequence of errors from behind. *sca* deletion at the stage of cluster formation in small clones should result in smaller clusters, if indeed Sca is an activator.

In figures 5b we show a quantification example of this phenotype, focusing on the small clones that appear in the furrow and therefore do not allow error propagation. For this analysis, we compared cluster sizes using the Imaris imaging processing software, which integrates different z-stacks and enables identifying all stained nuclei, even if present in different Z-planes. Calculating the volume of a cluster, rather than its area, is a more reliable method for obtaining cluster sizes, as in many cases some of the nuclei are in different plains and calculating surfaces would lead to an incorrect assessment. The results clearly show that depletion of sca in shallow clones results in smaller clusters, as expected if Sca functions as an activator of $\boldsymbol{ato}$ expression (Figure 5e and 5j). Figure 5d shows a large clone of $\boldsymbol{sca^-}$ that spans several columns of cells behind the MF, allowing noise to propagate and excess cells to be chosen.

Supplementary Note 10: The Egfr phenotype is not a result of apoptosis in the MF

Previous studies have shown that loss of Egfr leads to impaired recruitment of non-R8 photoreceptors and increased apoptosis behind the MF [11]. To make sure that the convergence of clusters in the MF in *pnt* mutants, implying Egfr signaling mediates an inhibitory signal at this stage, is not a result of increased cell death, we stained *pnt* mutants for cleaved caspase to mark apoptosis and for DAPI (Supplementary Figure 5a and 5b). As expected, *pnt* depletion increases apoptosis ahead and behind the MF in comparison to wild type. In sharp contrast, no apoptosis is observed in the region of the MF in which the clusters are formed. Further, we find no apparent change in the density and morphology of cells in the in *pnt* mutants compared to WT. These results confirm that the closer cluster spacing in the *pnt* mutant does not results from increased cell death but reflects the role of Egfr in inhibiting *ato* expression, as previously suggested [10].

Supplementary Note 11: Robustness is improved by cell constriction at the morphogenetic furrow

Our simulations also indicated that noise buffering is significantly improved when clusters are tightly packed (Figures 1d and 3e). This is because more cells are now positioned in the immediate vicinity of the inhibitor-secreting cell. Inhibitor diffusion can therefore be reduced, while maintaining inhibition of a sufficient number of cells, thereby reducing sensitivity to perturbations. We hypothesized that apical tissue indentation at the MF, which accompanies cluster formation, may function to effectively increase the

number of close neighbors, and thereby support robustness. The plausibility of the model was indeed confirmed by our simulations (Supplementary Figure 6a-6d).
To test this hypothesis, we examined clones depleted of *Rho kinase* (*rok*) RNA, which functions downstream of *hh* and is required for the apical constriction of cells [12]**.** For preventing constriction in the furrow, *rok* knockdown clones were generated in *hs-Flp*; *UAS-rok* KK107802/+; *actin5C-FRT-CD2-FRT-GAL4>FRT, UAS-GFP*/+ larvae, by exposing *hs-flp*; *rok-RNAi*; *act-GAL4* 1st instar larvae to a 20 minute heat pulse at 37°C. As predicted, cluster formation was significantly perturbed in such clones. In some cases, we observed single cells rather than clusters, while in others abnormally large clusters were detected (Supplementary Figure 6e and 6f). These distinct phenotypes can be explained by the time during which constriction was perturbed, relative to cluster formation. Note that the furrow-associated constriction extends anterior to the MF and may therefore facilitate robustness also in cases where cluster formation precedes the furrow (Supplementary Figure 6g).

Supplementary Methods

Quasi steady-state approximation for $h(x,y,t)$:

We describe the approximated dynamics of h we use in our simulations using the same assumptions and calculations previously described in Notes 1 and 2. h is a long-range activator. It is produced and secreted by selected cells expressing $a > a_h$ and diffuses away from those cells, to initiate a production at anterior, not yet patterned regions. A precise solution for h is difficult, since the producing cells at each time point are scattered according to earlier dynamics. Furthermore, non-R8 photoreceptors contribute to h propagation. Since we are interested in a symmetric pattern that is built up sequentially, we replace the precise term for h production by a uniform production posterior to the furrow:

(12.1)
$$\tau_h \frac{dh}{dt} = P_h \theta(vt - y) + D_h \frac{\partial^2 h}{\partial y^2} - h$$

Here y is the position along the anterior-posterior axis, and v is the velocity by which the differentiation wave propagates. Considering the periodic dynamics described above, we can assume that v is constant in time. In general, h is solved in a self-consistent manner, depending on the rate by which new cells are being selected and begin producing h.

We examine for solutions whereby h propagates with a velocity v, so that at each time point $h(y,t) = h(y - vt)$. Denoting $\bar{y} = y - vt$ we can write:

(12.2)
$$\frac{dh}{dt} = D_h \frac{\partial^2 h}{\partial \bar{y}^2} + v\tau_h \frac{\partial h}{\partial \bar{y}} - h + P_h \theta(-\bar{y})$$

Whose quasi-steady state solution is given by:

(12.3)

$$h(y,t) = \begin{cases} P_h - P_h\left(\frac{v\tau_h + c_1}{2c_1}\right) exp\left[\left(\frac{-v\tau_h + c_1}{2D_h}\right)\bar{y}\right] & \bar{y} < 0 \\ P_h\left(\frac{-v\tau_h + c_1}{2c_1}\right) exp\left[\left(\frac{-v\tau_h - c_1}{2D_h}\right)\bar{y}\right] & \bar{y} > 0 \end{cases}$$

$$c_1 = \sqrt{(v\tau_h)^2 + 4D_h}$$

Here $\bar{y} > 0$ corresponds to the position anterior to the differentiation wave that has not differentiated. $\bar{y} < 0$ corresponds to the posterior position that has differentiated already. In this region, h is approximately constant, decaying slowly close to the differentiation wave (Supplementary Figure 2c).

Numerical solution of the full model

Here we first refer to the simulations described in Figures 1d, 1e and 3c in the main text and to all similar phase diagrams in the SI. All simulations were held on a grid of 42X42 cells packed as described below. Equations (1.1) and (6.1) were solved discretely, in time steps of 10^{-2} , using a custom-written Matlab program implementing an explicit forward Euler method. The equation for h was not solved explicitly, but we assumed that h travels as a sweeping wave with speed v, as described by Eq. (12.3). All other parameters were defined by the numerical solutions of the equations above. As the resulting pattern depends on the initial conditions, in each simulation we tried all possibilities for initial cluster spacing, and report the maximal noise allowed for optimal initial spacing. Reflecting boundary conditions were assumed.

For simulations of the complete model, described in Note 7 and in Figure 4, a custom-written Matlab program implementing an explicit Euler method solved Eq. (7.1). A band of Notch (N) and of activated Notch (N_δ) was initiated in the position of the MF, that in our simulation contained three columns of cells. Levels of N and N_δ in columns ahead of the MF were also each time initiated to some different values (See parameters below). The levels of δ sensed by each cell is the sum of δ expressed by all of its neighboring cells.

2D packing

We assumed a 2D packing of 42X42 cells for clusters of up to 10 cells and of 60*60 for clusters of more than 10 cells, in which six neighbors surround each cell. Such packing was obtained using Voronoi diagrams, as implemented in Matlab.

Adding Noise in the full model simulations

In all phase diagrams appearing in the main text (in Figures 1d, 1e and 3c) and in the SI, noise was added to the production constants of a, u and s by choosing the constants out of a uniform distribution $P \to [P - P \cdot \text{noise}, P + P \cdot \text{noise}]$.

For a cell i surrounded by 6 neighbors (with indexes j), the diffusion term for u (and similarly for s) is given by Laplacian operator $D_u \nabla^2 u = \sum_j D_u^{i,j} \left(u^j - u^i\right)$, where

$D_u^{i,j} = D_u^{j,i}$ is the diffusion exchange rate between two neighboring cells. To capture variability in cell size, we added noise also to the diffusion constants in the same manner, while keeping $D_u^{i,j} = D_u^{j,i}$. This is equivalent to assuming differences in cell size, as the diffusion defines the relevant radius on which inhibition (or activation) is relevant. In Supplementary Figure 1 and 3 noise was added to all parameters, except to parameters that determine h propagation.

Defining pattern failure in the full model simulations

In the diagram phase shown in Figure 1d, a pattern was considered destroyed if an elongated-shaped cluster of more than 4 cells case or more than 5% cases of 'twinning' occurred (two cells or more differentiated adjacently). In the diagram phase shown in Figures 1e and 3c, a pattern was considered destroyed if an elongated cluster with more than 10 cells formed.

The same definitions hold for the corresponding Supplementary Figure 1, 3 and 6.

In the diagram phase shown in Figure 4, a pattern was considered destroyed if a cluster of more than 10 cells was formed or if more than 5% cases of twining occurred.

Numerical solution of the simplified model

Here we refer to the simulations described in Figures 2b and 2d in the main text. Eq. (3.9) generally describes an inhibition circle which is dependent on cluster size and on other properties of the inhibitor u. In these simulations each pattern was characterized by two parameters: cluster size and the inhibition radius R. In each simulation, an initial column of evenly spaced clusters was placed at the beginning of a grid, around which centers inhibition radiuses of size R were obtained. From here, we let the system propagate, where cells were allowed to differentiate in n columns out of the inhibitory circles. Reflecting boundary conditions were assumed. All possibilities for the initial spacing of the clusters were tried, and the noise shown is for the optimal initial spacing allowing maximal noise. 10,000 samples of R were evenly taken from the domain $\mathrm{R} = [1,12]$ for each cluster size. The activation radiuses of s in Figure 2d were chosen for each cluster according to some arbitrary distribution given by solving Eq. (3.9) for $P_s = 2, D_s = 0.75, s_1 = 0.08$ for clusters ranging from 1 to 20 cells.

Adding spatial noise to the simplified model simulations

In our simulations the noise describes the percentage of variability between clusters given an inhibition radius R. Noise was added to the radiuses in the following manner: for each cluster, R was chosen out of a uniform distribution $\mathrm{R} \rightarrow [\mathrm{R} - \mathrm{R} \cdot \mathrm{noise}, \mathrm{R} + \mathrm{R} \cdot \mathrm{noise}]$. In opposed to Eq. (3.9), we assumed that the inhibition radius is not affected by fluctuations in cluster size. Adding this dependency would have further increased noise sensitivity.

Defining pattern failure in the simplified model simulations

A pattern of cluster size 1 was considered destroyed when a cluster of size larger than 3 was formed. Similarly, a cluster of 3, 6, and 10 were considered destroyed when clusters of 6, 10 and 15 formed respectively. All simulations were run until cell differentiation reached the end of the grid.

Parameters

Most of the parameters were chosen to be consistent with the parameters in the prevailing model [1], with the following modifications:

i) The hill-functions (with the hill coefficients n=8) used in the previous study, were replaced by step-functions. This is practically the same, but enabled analytical solutions of some aspects of the dynamics.

ii) We chose a somewhat higher diffusion coefficient of Hh ($D_h = 600$ instead of $D_h = 200$). As long as this value is high, this choice does not affect the dynamics of cluster formation (Eq. 12.3).

iii) The eye disc has ~2000 cells. All simulations in our work were therefore performed on large grids containing at least 42X42 cells, which approximate the eye disc and give noise proper opportunity to propagate.

Parameters for the simulations in Figure 1d appear in Supplementary Table 1. 50 values of D_u and 400 values of u_1 were sampled in log-space from the given domains. For simulations in Figure 1e we used the same parameters as in Supplementary Table 1 except for $v = 0.75$.

Parameters for the simulations in Figure 3c were the same as in Supplementary Table 1, except for the parameters that appear in Supplementary Table 2.

Parameters for the simulations in Figure 4 were the same as in Supplementary Table 1 and 2, except for the parameters that appear in Supplementary Table 3. We set the levels of N and N_δ to initially be 0.15 and 0.09 in the MF, respectively. Ahead of the MF we set the levels of N and N_δ to 1.5 and 1, respectively.

Normalizing noise in inhibitor production

As is explained in main text, most sources of patterning failure are due to fluctuations in inhibition radiuses that lead to catastrophes. This is directly observed in the simulations in Figure 2b, where noise was added directly to the inhibition radiuses themselves. In the full simulations, noise in the inhibition radiuses was included indirectly, through the effect of the different molecular parameters (e.g. inhibitor diffusion coefficient or production rate) on these inhibition radiuses. We noted that in conditions where the threshold for inhibition u_1 is very low (compared to u production), the inhibition radius becomes rather insensitive to u production rate [13]. In this limit, even very low levels of u are sufficient for inhibition. This limit, however, is problematic in the sense that when the inhibition threshold is very low, the mean number of signaling (inhibitor) molecules that are sensed at this regions are very low and therefore more susceptible to random (poissonian) fluctuations. The expected fluctuations in this readout scale, with the number of molecules required for this inhibition u_1 is $\frac{1}{\sqrt{u_1}}$. We therefore normalized the noise in production rate by multiplying it by $\sqrt{\frac{u_1}{u_1^{min}}}$, where $u_1^{min} = 2 \cdot 10^{-2}$ as seen in Supplementary Table 1.

Considering constriction

Following the observation that noise buffering is largely improved when clusters are tightly packed as seen in Figures 1d, 1e, 2b, 2d and 4c, we hypothesized that tissue

indentation, which accompanies cluster formation, may function to effectively increase the number of close neighbors and thereby increase robustness. Accordingly, in the simulations shown in Supplementary Figure 6, we assumed the cells in the clusters can reach by immediate diffusion also cells that are places two columns ahead, and not only close neighbors, although with a lowered diffusion rate. Eqs. (1.1) and (6.1) were solved as described above, for cases of clusters of one cell and of up to seven cells, respectively. Diffusion of u and s was now allowed to reach beyond the six immediate neighbors of each cell, one column ahead, with a lower rate of 80% relative to the values in Supplementary Tables 1 and 2. We assumed reduced protein production by the same rate of 80%. Such simulations demonstrate that accounting for cell constriction in this manner significantly improves robustness.

Supplemental References

1. Lubensky, D. K., Pennington, M. W., Shraiman, B. I. & Baker, N. E. A dynamical model of ommatidial crystal formation. *Proc. Natl. Acad. Sci. U. S. A.* **108,** 11145–11150 (2011).

2. Spratford, C. M. & Kumar, J. P. Extramacrochaetae imposes order on the Drosophila eye by refining the activity of the Hedgehog signaling gradient. *Development* **140,** 1994–2004 (2013).

3. Powell, P. A., Wesley, C., Spencer, S. & Cagan, R. L. Scabrous complexes with Notch to mediate boundary formation. *Nature* **409,** 626–30 (2001).

4. Lee, E. C., Yu, S. Y. & Baker, N. E. The scabrous protein can act as an extracellular antagonist of notch signaling in the Drosophila wing. *Curr. Biol.* **10,** 931–4

5. Barad, O., Rosin, D., Hornstein, E. & Barkai, N. Error minimization in lateral inhibition circuits. *Sci. Signal.* **3,** ra51 (2010).

6. Greenwood, S. & Struhl, G. Progression of the morphogenetic furrow in the Drosophila eye: the roles of Hedgehog, Decapentaplegic and the Raf pathway. *Development* **126,** 5795–808 (1999).

7. Baonza, A. & Freeman, M. Notch signalling and the initiation of neural development in the Drosophila eye. *Development* **128,** 3889–98 (2001).

8. Hu, X., Lee, E. C. & Baker, N. E. Molecular analysis of scabrous mutant alleles from Drosophila melanogaster indicates a secreted protein with two functional domains. *Genetics* **141,** 607–17 (1995).

9. Ellis, M. C., Weber, U., Wiersdorff, V. & Mlodzik, M. Confrontation of scabrous expressing and non-expressing cells is essential for normal ommatidial spacing in the Drosophila eye. *Development* **120,** 1959–69 (1994).

10. Baonza, A., Casci, T. & Freeman, M. A primary role for the epidermal growth factor receptor in ommatidial spacing in the Drosophila eye. *Curr. Biol.* **11,** 396–404 (2001).

11. Fan, Y. & Bergmann, A. Multiple Mechanisms Modulate Distinct Cellular Susceptibilities toward Apoptosis in the Developing Drosophila Eye. *Dev. Cell* **30,** 48–60 (2014).

12. Corrigall, D., Walther, R. F., Rodriguez, L., Fichelson, P. & Pichaud, F. Hedgehog signaling is a principal inducer of Myosin-II-driven cell ingression in Drosophila epithelia. *Dev. Cell* **13,** 730–42 (2007).

13. Eldar, A., Rosin, D., Shilo, B.-Z. & Barkai, N. Self-enhanced ligand degradation underlies robustness of morphogen gradients. *Dev. Cell* **5,** 635–46 (2003).
